# Homeostatic Shrinkage of Dendritic Spines Requires Melatonin Type 3 Receptor Activation During Sleep

**DOI:** 10.1002/advs.202400253

**Published:** 2024-08-09

**Authors:** Shiyin Li, Xin Li, Minmin Lu, Quanhui Chen, Di Yao, Xiaoqian Yu, Zhen Li, Woo‐ping Ge, Na Wang, Jiehua Jin, Yaling Wang, Yixiang Liao, Fenlan Luo, Jie Yan, Xuedan Chen, Chenggang Jiang, Faguo Yue, Dong Gao, Xiangdong Tang, Hong Guo, Yanjiang Wang, Xiaowei Chen, Jianxia Xia, Min Xu, Shuancheng Ren, Chao He, Zhian Hu

**Affiliations:** ^1^ Department of Physiology Institute of Brain and Intelligence Third Military Medical University Chongqing 400038 China; ^2^ School of Basic Medical Sciences and IDG/McGovern Institute for Brain Research Tsinghua University Beijing 100084 China; ^3^ School of Basic Medical Sciences Capital Medical University Beijing 100069 China; ^4^ Chinese Institute for Brain Research Beijing 102206 China; ^5^ Department of Medical Genetics College of Basic Medical Sciences Third Military Medical University Chongqing 400038 China; ^6^ Department of Sleep and Psychology Chongqing Health Center for Women and Children Chongqing 401147 China; ^7^ Sleep and Psychology Center Bishan Hospital of Chongqing Medical University Chongqing 402760 China; ^8^ Department of Sleep and Psychology The Fifth People's Hospital of Chongqing Chongqing 400062 China; ^9^ Sleep Medicine Center Laboratory of Anaesthesia and Critical Care Medicine Translational Neuroscience Center West China Hospital Sichuan University Chengdu 610041 China; ^10^ Department of Neurology Daping Hospital Third Military Medical University Chongqing 400042 China; ^11^ Chongqing Institute for Brain and Intelligence Guangyang Bay Laboratory Chongqing 400064 China; ^12^ Brain Research Center Institute of Brain and Intelligence Third Military Medical University Chongqing 400038 China; ^13^ Institute of Neuroscience，Center for Excellence in Brain Science and Intelligence Technology Chinese Academy of Sciences Shanghai 200031 China

**Keywords:** medial entorhinal cortex, melatonin, sleep, spatial memory

## Abstract

High‐frequency oscillatory activity in cognition‐related neural circuits during wakefulness consistently induces the growth of dendritic spines and axonal terminals. Although these structural changes are essential for cognitive functions, it is hypothesized that if these newly expanded structures fail to establish functional connections, they may become superfluous. Sleep is believed to facilitate the reduction of such redundant structures to maintain neural homeostasis. However, the mechanisms underlying this pruning process during sleep remain poorly understood. In this study, that melatonin type 3 receptors (MT_3_Rs) are selectively expressed in the stellate neurons of the medial entorhinal cortex (MEC) is demonstrated, an area where high melatonin levels are detected during sleep. Activation of MT_3_Rs during sleep initiates the shrinkage of dendritic spines in stellate neurons by downregulating neural network activity and dephosphorylating synaptic proteins in the MEC. This process is disrupted when MT_3_R expression is knocked down or when MT_3_Rs are blocked during sleep. Notably, interference with MT_3_Rs in the MEC during sleep impairs the acquisition of spatial memory but does not affect object memory acquisition following sleep. These findings reveal novel molecular mechanisms involving melatonin and MT_3_Rs in the regulation of dendritic spine shrinkage during sleep, which is crucial for the acquisition and consolidation of spatial memory.

## Graphical abstract

Sleep is thought to promote the shrinkage of unnecessary synaptic structures to maintain homeostasis. However, the specific mechanisms underlying this phenomenon during sleep are still not well understood. This study uncovers novel molecular mechanisms that involve melatonin/MT_3_Rs in the shrinkage of dendritic spines during sleep, which contributes to the acquisition and consolidation of spatial memory.

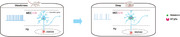

## 1 Introduction

High‐frequency network oscillations in cognition‐related neural circuits during wakefulness induce significant structural changes, such as an increase in the number and volume of dendritic spines and axonal terminals. These changes lay the foundation for the formation of new synapses, which are crucial for cognitive functions like memory storage.^[^ ^1^, ^2^, ^3^ ^]^ However, many of these altered dendritic spines and axonal terminals may fail to establish functional synaptic connections, rendering them redundant. The accumulation of these surplus structures occupies space and decreases the brain's signal‐to‐noise ratio during wakefulness, leading to an unsustainable state.^[^ ^4^, ^5^, ^6^ ^]^ Sleep has been reported to facilitate the reduction of these extra dendritic spines and axonal terminals in the drosophila brain, as well as in the primary motor and somatosensory cortex of rodents. This pruning process is essential for maintaining structural homeostasis and is closely linked to improved cognitive performance following sleep.^[^ ^4^, ^6^, ^7^, ^8^, ^9^, ^10^ ^]^ Despite this, the mechanisms underlying synaptic structure shrinkage during sleep remain poorly understood.

Melatonin, secreted by the pineal gland and regulated by the light/dark cycle, begins to rise as darkness falls and peaks between 2:00 A.M. and 4:00 A.M.^[^ ^11^, ^12^, ^13^ ^]^ This secretion pattern suggests that melatonin might have additional functions during sleep beyond its role in sleep induction. Given that synaptic structure shrinkage is a key function of sleep, melatonin may mediate this process to maintain brain homeostasis.

Melatonin's primary mechanism of action in the brain involves three distinct receptor types. Melatonin type 1 receptors (MT_1_Rs) and type 2 receptors (MT_2_Rs) are classical G‐protein‐coupled receptors.^[^ ^14^ ^]^ Additionally, melatonin type 3 receptors (MT_3_Rs), also known as quinone reductase 2, are considered putative intracellular receptors for melatonin.^[^ ^15^ ^]^ MT_3_Rs have been reported to decrease neuronal activity in the hippocampus and insular cortex,^[^ ^16^, ^17^, ^18^ ^]^ though their physiological role remains unclear. In this study, we identify that MT_3_Rs are selectively expressed in the stellate neurons of the medial entorhinal cortex (MEC), a region that serves as a gateway to the hippocampus and is critically involved in spatial memory.^[^ ^19^ ^]^ Using high‐resolution high‐performance liquid chromatography‐mass spectrometry (HPLC‐MS), we detect high melatonin levels in the MEC during sleep. We show that MT_3_R activation in the MEC during sleep drives the shrinkage of dendritic spines in stellate neurons by downregulating neural network activity and dephosphorylating synaptic proteins. Furthermore, we demonstrate that knocking down or blocking MT_3_Rs in the MEC during sleep impairs spatial memory acquisition and consolidation. Our findings uncover novel molecular mechanisms underlying dendritic spine shrinkage during sleep, highlighting the role of melatonin and MT_3_Rs in maintaining neural homeostasis.

## 2 Results

### 2.1 MT_3_Rs are Specifically Expressed in Stellate Neurons of MEC Where High Levels of Melatonin are Detected During Sleep

**Figure 1:**
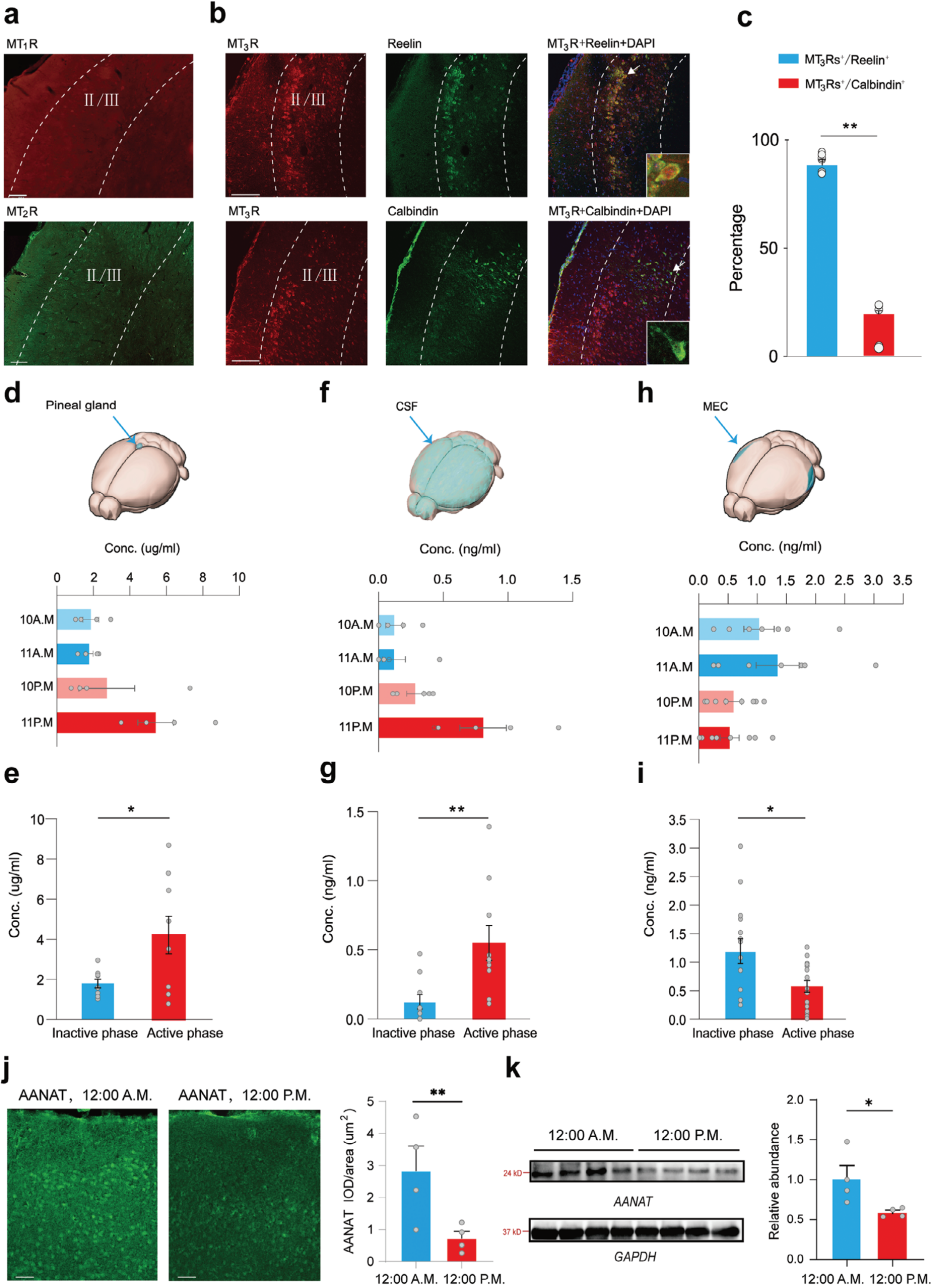
Selective expression of MT_3_Rs in MEC stellate neurons and elevated levels of melatonin within the MEC during the inactive phase. a) Representative images showing the expression of MT_1_Rs and MT_2_Rs in the MEC. Scale bar: 100 µm. b) Representative images showing the expression of MT_3_Rs in the reelin^+^ stellate neurons and calbindin^+^ pyramidal neurons of the MEC. Scale bar: 100 µm. c) Percentage of reelin^+^ stellate neurons and calbindin^+^ pyramidal neurons that were positive for MT_3_Rs (mean ± SEM). Mann‐Whitney Rank Sum Test, **P < 0.01, n = 6 sections from 3 rats. d) Concentration of melatonin in the pineal gland tissue at 4 time points (10 A.M. and 11 A.M. represent inactive phase, 10 P.M. and 11 P.M. represent active phase). e) Bar graphs with overlaid dot plots showing the average concentration of melatonin in the pineal gland tissue during the day (10 A.M. and 11 A.M.) and night (10 P.M. and 11 P.M.) (mean ± SEM). Welch's t‐test, *P < 0.05, n = 9 rats for each group. f) Concentration of CSF melatonin taken through the foramen magnum at 4 time points (10 A.M., 11 A.M., 10 P.M., and 11 P.M.). g) Bar graphs with overlaid dot plots showing the average concentration of the CSF melatonin during the day (10 A.M. and 11 A.M.) and night (10 P.M. and 11 P.M.) (mean ± SEM). Mann‐Whitney Rank Sum Test, **P < 0.01, n = 10 rats for each group. h) Melatonin measurements in tissue homogenates of the MEC at 4 time points (10 A.M., 11 A.M., 10 P.M., and 11 P.M.). i) Bar graphs with overlaid dot plots showing the average concentration of melatonin in the MEC tissue homogenates during the day (10 A.M. and 11 A.M.) and night (10 P.M and 11 P.M.) (mean ± SEM). Welch's t‐test, *P < 0.05, n = 15 sample points, inactive phase, n = 16 sample points, active phase, each sample point consists of bilateral MEC tissue samples from 3 rats. j) Images showing AANAT expression levels in the MEC (left), Scale bar: 50 µm. Bar graphs with overlaid dot plots showing the AANAT expression levels at active and inactive phases (mean ± SEM). Mann‐Whitney Rank Sum Test, **P < 0.01, n = 12 sections from 4 rats, 12 A.M., n = 12 sections from 4 rats, 12 P.M. (right). k) Immunoblot analysis of AANAT expression levels at active and inactive phase (mean ± SEM). Student's t‐test, *P < 0.05, n = 4 rats, 12 A.M., n = 4 rats, 12 P.M. MT_1_Rs, Melatonin type 1 receptors; MT_2_Rs, Melatonin type 2 receptors; MT_3_Rs, Melatonin type 3 receptors; MEC, medial entorhinal cortex; CSF, cerebrospinal fluid; AANAT, aralkylamine N‐acetyltransferase; GAPDH, glyceraldehyde‐3‐phosphate dehydrogenase.

The MEC displays significant circadian variations in neuronal activity, characterized by high‐frequency oscillatory activities such as theta (4–12 Hz) and gamma (25–120 Hz) oscillations during wakefulness.^[^ ^20^, ^21^ ^]^ This suggests a heightened need for sleep‐induced homeostatic restoration of synaptic structures in the MEC. We investigated the expression patterns of melatonin receptors in this brain region of rats. Our results revealed that MT_1_Rs and MT_2_Rs, the classical G‐protein‐coupled receptors,^[^ ^14^ ^]^ were expressed at relatively low levels in the MEC (**Figure** **1**a). MT_3_Rs, which have been reported to downregulate neuronal network excitability,^[^ ^16^, ^17^, ^18^ ^]^ were of particular interest. Immunofluorescence and fluorescence in situ hybridization (FISH) analyses showed that MT_3_Rs were highly expressed in the superficial layers of the MEC. These MT_3_Rs were predominantly found in reelin^+^ stellate neurons, which constitute the perforant pathway and serve as the primary mediators of information transmission from the MEC to the hippocampus.^[^ ^22^ ^]^ In contrast, MT_3_Rs were sparsely expressed in calbindin^+^ pyramidal neurons, another type of excitatory projection neuron located in the MEC (Figure 1b,c; Figure S1a–c, Supporting Information). Quantitative reverse transcription‐polymerase chain reaction (RT‐qPCR) and immunoblot analysis further confirmed that MT_3_Rs were significantly more abundant than MT_1_Rs and MT_2_Rs in the MEC (Figure S1d–g, Supporting Information).

We next examined whether high concentrations of melatonin are present in the MEC to act on MT_3_Rs during sleep in the light phase of rats. Previous studies have shown that the pineal gland releases melatonin into the cerebrospinal fluid (CSF) and bloodstream, with its secretion regulated by the light/dark cycle. Melatonin levels in the CSF and blood are typically low during the light phase in both nocturnal and diurnal animals, including rodents.^[^ ^11^, ^12^, ^13^, ^23^, ^24^ ^]^ Using HPLC‐MS, we measured melatonin levels in the pineal gland and CSF of rats during the light (10:00 A.M. and 11:00 A.M.) and dark phases (10:00 P.M. and 11:00 P.M.). Rats were predominantly asleep during the light phase and awake during the dark phase, as confirmed by live video recordings. Consistent with previous studies,^[^ ^23^, ^24^, ^25^ ^]^ melatonin levels in the pineal gland and CSF were lower during the light phase than the dark phase (Figure 1d–g).

Interestingly, despite high melatonin levels in the pineal gland and CSF during the dark phase, other reports suggest that melatonin has a sleep‐promoting effect specifically during the light phase in rodents.^[^ ^26^, ^27^ ^]^ This discrepancy could be explained by extrapineal production of melatonin. Melatonin can be produced by various cells, leading to different secretion patterns.^[^ ^28^ ^]^ For instance, in the rat cerebral cortex, cytosolic melatonin peaks during the light phase (11:00 A.M. to 12:00 P.M.).^[^ ^29^ ^]^ Based on this, we measured melatonin content in MEC tissue. Remarkably, melatonin levels in MEC tissue homogenates were 110% higher during the light phase compared to the dark phase (Figure 1h,i). Additionally, we detected the expression of aralkylamine N‐acetyltransferase (AANAT), the enzyme responsible for melatonin synthesis, in the MEC. AANAT expression was high during sleep and significantly reduced during wakefulness (Figure 1j,k). These findings suggest that, in addition to the pineal gland, other brain regions, including the MEC, can endogenously secrete melatonin independently of the light/dark cycle. This extrapineal production of melatonin results in elevated melatonin levels during the light phase in specific brain regions, enabling its involvement in sleep‐related functions.

### 2.2 Shrinkage of Dendritic Spines in MEC Stellate Neurons Requires MT_3_R Activation During Sleep

Given the high expression of MT_3_Rs in stellate neurons and the elevated levels of melatonin in the MEC during the light phase, we investigated whether melatonin signaling during sleep is necessary for the shrinkage of dendritic spines in these cells. Previous studies have shown that sleep promotes the homeostatic shrinkage of dendritic spines and axon‐spine interfaces in the primary motor and somatosensory cortex of rodents.^[^ ^4^, ^8^ ^]^ However, evidence of synaptic structural shrinkage in the memory‐related MEC during sleep is limited.

**Figure 2:**
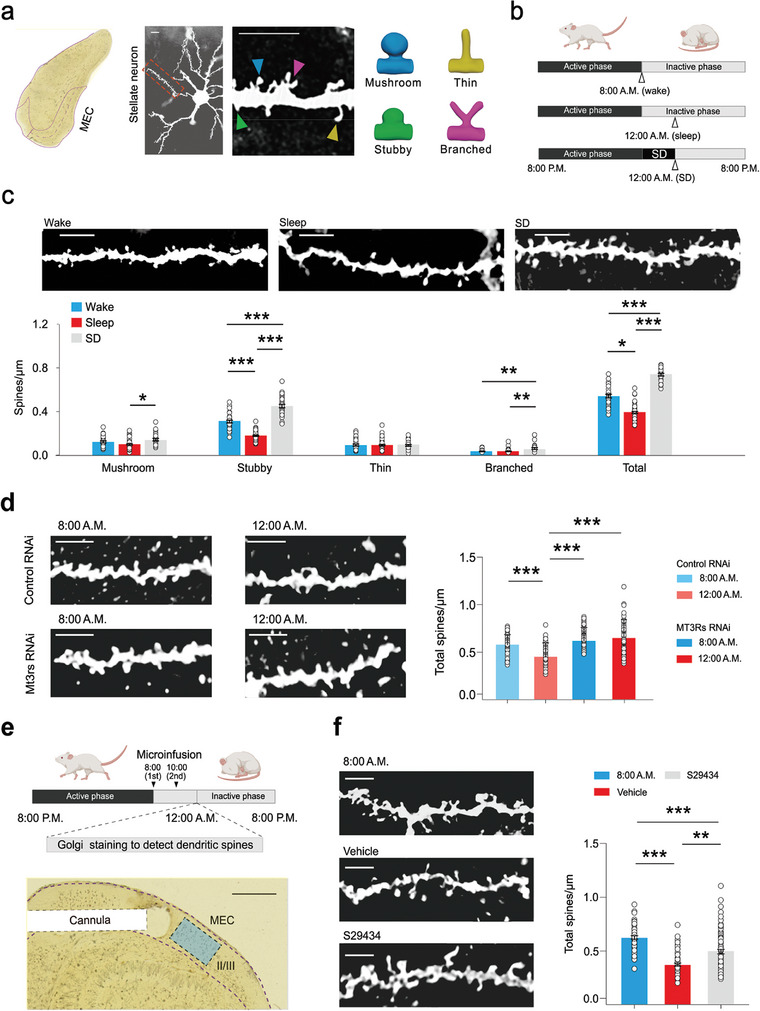
MT_3_R activation during sleep is necessary for the shrinkage of dendritic spines in MEC stellate neurons. a) A representative stellate neuron in layer II/III of the MEC was identified and distinguished based on its morphology. The dendritic spines of the secondary dendrites in these neurons are counted (left). Different types of dendritic spines were classified by their morphology. Scale bar: 10 µm. b) Paradigm for detecting dendritic spines at the wake, sleep, and SD. Arrowheads indicate the time of superficial layers of MEC collection for Golgi staining. c) Dendritic spine density of the stellate neurons after wake, sleep, and SD. Scale bar: 10 µm. mushroom (mean ± SEM): Kruskal‐Wallis One Way Analysis of Variance on Ranks, *P < 0.05, n = 31 cells of wake, n = 56 cells of sleep, n = 28 cells of SD; stubby (mean ± SEM): Kruskal‐Wallis One Way Analysis of Variance on Ranks, ***P < 0.001, n = 31 cells of wake, n = 56 cells of sleep, n = 28 cells of SD; thin (mean ± SEM): Kruskal‐Wallis One Way Analysis of Variance on Ranks, *P* > 0.05, n = 31 cells of wake, n = 56 cells of sleep, n = 28 cells of SD; branched (mean ± SEM): Kruskal‐Wallis One Way Analysis of Variance on Ranks, **P < 0.01, n = 31 cells of wake, n = 56 cells of sleep, n = 28 cells of SD; total (mean ± SEM): Kruskal‐Wallis One Way Analysis of Variance on Ranks, *P < 0.05, ***P < 0.001, n = 31 cells of wake, n = 56 cells of sleep, n = 28 cells of SD. d) Dendritic spine density of the stellate neurons in MT_3_R RNAi and control groups (mean ± SEM). Scale bar: 10 µm. Kruskal‐Wallis One Way Analysis of Variance on Ranks, ***P < 0.001, n = 57 cells of control 8:00 A.M., n = 70 cells of control 12:00 A.M., n = 65 cells of RNAi 8:00 A.M., n = 80 cells of RNAi 12:00 A.M. e) Paradigm for detecting dendritic spines in layer II/III of MEC after blockade of MT_3_Rs by S29434 during sleep. Scale bar: 2 mm. f) Changes in the dendritic spine density of the stellate neurons after blockade of MT_3_Rs by S29434 during sleep (mean ± SEM). Scale bar: 10 µm. Kruskal‐Wallis One Way Analysis of Variance on Ranks, **P < 0.01, ***P < 0.001, n = 60 cells of 8:00 A.M., n = 80 cells of Vehicle, n = 79 cells of S29434. SD, sleep deprivation; RNAi, RNA interference.

To directly observe and assess alterations in dendritic spines of stellate neurons throughout the sleep‐wake cycle, we utilized Golgi staining in adult male rats. These neurons are characterized by an oval‐shaped soma with multiple thick primary dendrites radiating outward. Dendritic spines on the secondary dendrites were quantified and categorized into four subtypes: mushroom, stubby, thin, and branched spines (**Figure** **2**a). After 4 h of sleep, the density of stubby dendritic spines significantly decreased, while mushroom, thin, and branched spines were minimally affected. This reduction in stubby spine density resulted in a 27% decline in total spine density. Conversely, 4 h of sleep deprivation (SD) led to a significant 78% increase in dendritic spine density (Figure 2b,c).

We further analyzed the head diameter and length of dendritic spines. Sleep consistently reduced the head diameter and length of dendritic spines by 6% and 14%, respectively (Figure S2, Supporting Information). Following 4 h of SD, we observed increases in both the head diameter and length of dendritic spines. Previous studies showed that the axon‐spine interface decreased by ≈18% after sleep compared to wakefulness in primary motor and somatosensory cortices.^[^ ^8^ ^]^ In the hippocampus, the volume of spine heads was reduced by ≈40%^[^ ^4^ ^]^ in sharp wave ripple‐emitting brain slices, and SD increased spine density and volume by 25% in CA1 pyramidal neurons.^[^ ^30^ ^]^ These findings suggest that sleep significantly impacts synaptic shrinkage in the memory‐related hippocampus‐MEC circuits.

To determine whether melatonin/MT_3_R signaling is crucial for the shrinkage of dendritic spines in stellate neurons of the MEC, we employed MT_3_R RNA interference (RNAi)‐directed transcript knockdown. This was achieved by injecting pAAV‐U6‐shRNA (Nqo2)‐CMV‐EGFP‐WPRE into the MEC, with pAAV‐U6‐NC‐CMV‐EGFP‐WPRE expressing random RNA fragments as the control. FISH and RT‐qPCR confirmed a significant decrease in MT_3_R expression in the superficial layers of the MEC following RNAi knockdown (Figure S3a,b, Supporting Information).

In control rats injected with viruses expressing random RNA fragments, significant shrinkage of dendritic spines in stellate neurons of the MEC was observed after 4 h of sleep. The densities of stubby and thin dendritic spines were significantly reduced by sleep, while other dendritic spine densities remained unaffected. In contrast, MT_3_R knockdown resulted in a significant increase in dendritic spine length, with total dendritic spine density and head diameter remaining unchanged at 8:00 A.M. (Figure S3d–f, Supporting Information). Importantly, after MT_3_R knockdown, the density (Figure 2d; Figure S3c,d, Supporting Information), length, and head diameter of dendritic spines in stellate neurons remained high, without significant shrinkage after 4 h of sleep at 12:00 A.M. (Figure S3e,f, Supporting Information).

To further validate this phenomenon, we blocked MT_3_Rs during sleep. Administration of S29434 (50 µM) in the MEC of adult male rats prevented dendritic spine shrinkage during sleep. This was evidenced by a significant increase in dendritic spine density (Figure 2e,f), head diameter, and length of dendritic spines in stellate neurons compared to control rats with 4 h of sleep (Figure S4, Supporting Information). Specifically, the densities of stubby and thin dendritic spines increased significantly following MT_3_R inhibition, while mushroom and branched spines were unaffected. This effect was not due to indirect impacts of MT_3_R inhibition on sleep/wake behavior, as S29434 administration did not alter sleep duration during the first 4 h of the light phase compared to the control group (Figure S5, Supporting Information).

We next examined whether melatonin‐induced dendritic spine shrinkage also occurs in female and aged rats. In adult female rats, dendritic spines of MEC stellate neurons shrank during sleep, dependent on MT_3_R activation. Blocking MT_3_Rs with S29434 significantly increased dendritic spine density following 4 h of sleep (Figure S6, Supporting Information). However, in aged male rats (21 months old), there was no significant reduction in dendritic spine density after 4 h of sleep compared to wakefulness, indicating impaired dendritic spine shrinkage during sleep. Additionally, aged rats exhibited a notable decrease in overall dendritic spine density compared to adult rats, suggesting an age‐related loss of dendritic spines in the MEC (Figure S7, Supporting Information).

### 2.3 MT_3_R Activation During Sleep Mediates Shrinkage of Dendritic Spines by Downregulating Neural Activity and Synaptic Protein Phosphorylation

**Figure 3:**
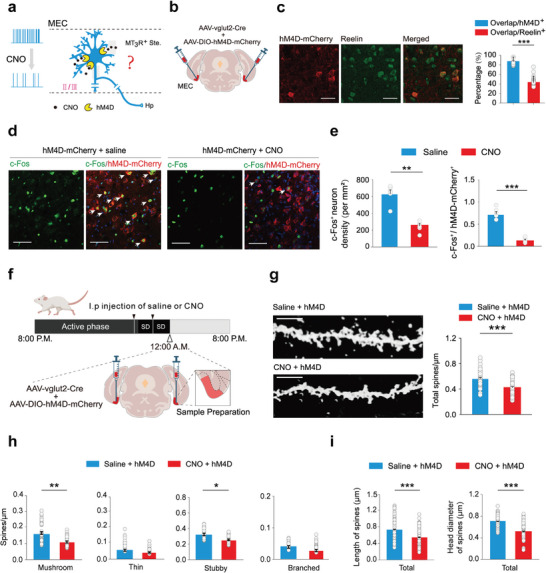
Downregulation of stellate neuron activity induces shrinkage of dendritic spines. a) An experimental paradigm showing whether downregulation of MT_3_R^+^ stellate neuron activity could drive the shrinkage of dendritic spines. Ste, stellate neuron. b,c) Selective expression of hM4D‐mCherry in reelin^+^ stellate neurons in the MEC (mean ± SEM). Mann‐Whitney Rank Sum Test, ***P < 0.001, n = 20 sections from 4 rats. Scale bar: 20 µm. d) Images showing the c‐Fos expression in the superficial layers of the MEC after saline and CNO injection. Scale bar: 50 µm. e) Quantitative analysis of c‐Fos expression level in the superficial layers of the MEC after saline and CNO injection. left (mean ± SEM), Mann‐Whitney Rank Sum Test, **P < 0.01, n = 6 sections from 3 rats; right (mean ± SEM), Student's t‐test, ***P < 0.001, n = 6 sections from 3 rats. f) Paradigm for detecting dendritic spines after selective inhibition of MT_3_R^+^ stellate neurons. g) Changes in the dendritic spine density of MT3R^+^ stellate neurons after chemogenetic inhibition (mean ± SEM). Scale bar: 10 µm, Mann‐Whitney Rank Sum Test, ***P < 0.001, n = 51 cells of saline, n = 37 cells of CNO. h) Alterations in the subtype dendritic spine density of the stellate neurons after inhibition of the stellate neuron activity mushroom (mean ± SEM), Mann‐Whitney Rank Sum Test, **P < 0.01, n = 51 cells of saline, n = 37 cells of CNO; thin (mean ± SEM), Mann‐Whitney Rank Sum Test, *P* = 0.564, n = 51 cells of saline, n = 37 cells of CNO; stubby (mean ± SEM), Mann‐Whitney Rank Sum Test, *P < 0.05, n = 51 cells of saline, n = 37 cells of CNO; branched (mean ± SEM), Mann‐Whitney Rank Sum Test, *P* = 0.080, n = 51 cells of saline, n = 37 cells of CNO. i) Changes in the length and head diameter of the dendritic spine of the stellate neurons after inhibition of the stellate neuron activity. length (mean ± SEM), Mann‐Whitney Rank Sum Test, ***P < 0.001, n = 1656 spines of saline, n = 865 spines of CNO; head diameter (mean ± SEM), Mann‐Whitney Rank Sum Test, ***P < 0.001, n = 1323 spines of saline, n = 726 spines of CNO. CNO, clozapine‐N‐oxide.

We next investigated how MT_3_R activation during sleep mediates the shrinkage of dendritic spines in MEC stellate neurons. Activity patterns in these neurons exhibit significant variation across the sleep‐wake cycle, characterized by heightened neural activity during wakefulness that facilitates information processing, and low neural activity, such as slow oscillations, during sleep.^[^ ^20^ ^]^ Since high neural activity during sensory stimuli and cognitive tasks induces activity‐dependent synaptic plasticity by increasing synapse volume and number,^[^ ^31^, ^32^, ^33^ ^]^ we hypothesized that reduced neural activity during sleep promotes the shrinkage of synaptic structures, including dendritic spines. To test this hypothesis, we examined the effects of chemogenetic inhibition of stellate neuron activity on dendritic spine shrinkage (**Figure** **3**a). A mixture of AAV2/9‐vglut2‐Cre and AAV2/9‐DIO‐hM4D‐mCherry was injected into the superficial layers of the MEC. Immunofluorescence confirmed selective expression of hM4D‐mCherry in reelin^+^ stellate neurons (Figure 3b,c). Intraperitoneal injection of clozapine‐N‐oxide (CNO, 1 mg kg^−1^) significantly reduced c‐Fos expression levels in the MEC, indicating effective inhibition of neural activity (Figure 3d,e). Remarkably, chemogenetic inhibition of stellate neuron activity during 4 h SD, while rats remained awake, led to a significant decrease in total dendritic spine density in stellate neurons (Figure 3f–h). The head diameter and length of dendritic spines also decreased significantly following this inhibition (Figure 3i).

Additionally, we selectively inhibited stellate neurons during the active phase without SD using the same chemogenetic approach. Similar to the SD condition, inhibition of stellate neuron activity during the active phase significantly reduced dendritic spine density, head diameter, and spine length (Figure S8, Supporting Information). These findings suggest that reduced stellate neuron activity is indeed associated with the shrinkage of dendritic spines.

**Figure 4:**
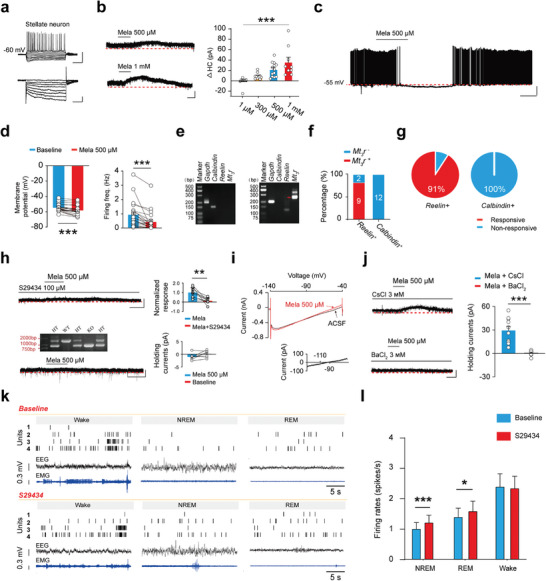
MT_3_R activation‐induced opening of potassium channels in stellate neurons exerts a down‐regulatory mechanism of neural activity during sleep. a) Electrophysiological properties of a stellate neuron. Top, Scale bar: 250 ms, 50 mV. Down, scale bar: 250 ms, 100 pA. b) Melatonin‐induced outward currents in the stellate neurons in a concentration‐dependent manner (mean ± SEM). Scale bar: 75 S, 40 pA. Kruskal‐Wallis One Way Analysis of Variance on Ranks, ***P < 0.001, n = 8 cells of 1 µM, n = 10 cells of 300 µM, n = 13 cells of 500 µM, n = 9 cells of 1 mM. c,d) Melatonin generated membrane hyperpolarization and inhibited neuronal firing frequency in the stellate neurons. Scale bar: 60 S, 20 mV. Membrane potential (mean ± SEM): Paired t‐test, ***P < 0.001, n = 23 cells; Firing freq (mean ± SEM): Wilcoxon Signed Rank Test, ***P < 0.001, n = 21 cells. e) The represented images of scRT‐PCR obtained from a pyramidal neuron (left) and a stellate neuron (right), respectively. f,g) scRT‐PCR showed that the majority of the stellate neurons (n = 9 cells in 11 cells) were positive for reelin and exhibited outward currents in response to melatonin (n = 10 cells in 11 cells, 91%). All the Calbindin^+^ pyramidal neurons were negative for MT_3_R and did not show outward currents in response to melatonin (n = 12 cells, 100%). h) Melatonin‐induced outward currents in the stellate neurons were blocked by MT_3_R antagonist S29434 (mean ± SEM). Scale bar: 100 S, 40 pA. Paired t‐test, **P < 0.01, n = 9 cells (top). Melatonin‐induced outward currents in the stellate neurons were abolished in MT_3_R knockout rats (mean ± SEM). Scale bar: 100 S, 40 pA. Wilcoxon Signed Rank Test, *P* = 0.297, n = 7 cells. (down). i) Reversal potentials of melatonin‐induced currents were close to the estimated theoretical K^+^ reversal potential (E_k_) by the Nernst equation. j) Melatonin‐induced currents were blocked by extracellular Ba^2+^, but not by Cs^+^ (mean ± SEM). Scale bar: 100 s, 40 pA. Welch's t‐test, ***P < 0.001. BaCl_2_: n = 10 cells, CsCl: n = 7 cells. k) EEG, EMG and unit recordings trace during wakefulness, NREM sleep, and REM sleep before and after blocking the MT_3_Rs. l) Average firing rate of MEC glutamatergic neurons in each state before and after blocking the MT_3_Rs. NREM (mean ± SEM): Wilcoxon Signed Rank Test, ***P < 0.001, n = 80 cells from 8 rats; REM (mean ± SEM): Wilcoxon Signed Rank Test, *P < 0.05, n = 80 cells from 8 rats; Wake (mean ± SEM): Wilcoxon Signed Rank Test, *P* = 0.650, n = 80 cells from 8 rats. Mela, Melatonin; scRT‐PCR, single‐cell Reverse Transcription‐Polymerase Chain Reaction; EEG, Electroencephalography; EMG, Electromyography; NREM sleep, Non‐Rapid Eye Movement sleep; REM sleep, Rapid Eye Movement sleep.

Next, we investigated whether melatonin/MT_3_R signaling could downregulate the activity of stellate neurons in the MEC. Using whole‐cell patch‐clamp recordings, we found that melatonin induced dose‐dependent outward currents in stellate neurons (**Figure** **4**a,b), hyperpolarized their membrane potentials, and inhibited their firing frequency (Figure 4c,d). In contrast, melatonin did not affect the holding currents (Figure S9a,b, Supporting Information) or firing frequency (Figure S9c,d, Supporting Information) of pyramidal neurons in the MEC. Single‐cell reverse transcription‐polymerase chain reaction (scRT‐PCR) combined with whole‐cell recording revealed that the majority of stellate neurons (91%, 9/11) were MT_3_Rs‐positive, and all MT_3_Rs‐positive neurons exhibited outward currents in response to melatonin. Conversely, pyramidal neurons, which were MT_3_Rs‐negative, showed no change in holding currents upon melatonin treatment (Figure 4e–g). These findings highlight a specific inhibitory role of melatonin on stellate neurons. This inhibitory effect was blocked by an MT_3_R antagonist and was absent in MT_3_R knockout rats (Figure 4h), but remained unaffected by an MT_1/2_R antagonist (Figure S9e, Supporting Information).

We further explored the ionic mechanisms underlying melatonin‐induced outward currents in stellate neurons. Although these neurons exhibit prominent inward I_h_ currents,^[^ ^34^ ^]^ melatonin did not affect the amplitude of these currents (Figure S9f, Supporting Information). Slow ramp command tests (dV/dt = −10 mV/s) were conducted to evaluate the *I–V* curves in the presence and absence of melatonin, revealing a reversal potential close to the estimated potassium equilibrium potential (E_k_) calculated using the Nernst equation (Figure 4i). Previous studies have shown that stellate neurons express high levels of two‐pore potassium channels sensitive to Ba^2+^ but not to Cs^+^.^[^ ^35^ ^]^ Consistent with this, the application of 3 mM Ba^2+^ in the extracellular fluid abolished melatonin's inhibitory effect, whereas Cs^+^ did not (Figure 4j).

The melatonin concentrations used in the MEC were in the micromolar range, higher than those effective in other brain regions where melatonin acts via classical G‐protein‐coupled MT_1_Rs and MT_2_Rs.^[^ ^36^ ^]^ To verify that this discrepancy was not due to experimental artifacts, we performed control experiments on glutamatergic neurons in the paraventricular nucleus of the thalamus (PVT), a wakefulness‐promoting area predominantly expressing MT_1_Rs and MT_2_Rs (Figure S10a, Supporting Information).^[^ ^37^ ^]^ Interestingly, nanomolar concentrations (50 and 100 nM) of melatonin inhibited these neurons in the PVT (Figure S10b–g, Supporting Information), suggesting that the differing effective concentrations might reflect variations in receptor characteristics and subcellular distribution.

We also investigated whether MT_3_R activation modulates neural network activity in vivo. Following established protocols,^[^ ^21^, ^37^ ^]^ we implanted a cannula for drug microinfusion and placed an electrode array for multi‐channel single‐unit recordings in the MEC's superficial layers (Figure S11a, Supporting Information). Blocking MT_3_Rs during sleep significantly reduced low‐frequency delta oscillation power while increasing the power of theta, beta, and gamma oscillations (Figure S11b,c, Supporting Information). Moreover, MEC excitatory neurons displayed the highest firing frequency during wakefulness, the lowest during non‐rapid eye movement (NREM) sleep, and an intermediate level during rapid eye movement (REM) sleep, indicating reduced stellate neuron activity during sleep (Figure 4k,l). MT_3_R blockade during the inactive phase significantly increased MEC neuron firing frequency during both NREM and REM sleep, without affecting wakefulness activity (Figure 4l). These findings demonstrate that MT_3_R activation suppresses MEC neuron firing during sleep.

To elucidate how MT_3_R activation and subsequent downregulation of neural activity during sleep induce dendritic spine shrinkage, we examined the involvement of protein kinases and synaptic protein phosphorylation, which are critical for the dynamic regulation of synaptic structures and the cytoskeleton. Protein kinase A (PKA) and protein kinase C (PKC) are known to modulate these processes through synaptic protein phosphorylation.^[^ ^33^, ^38^ ^]^ Previous research has shown that sleep reduces the activity of protein kinases and decreases synaptic protein phosphorylation, but the underlying mechanisms remain unclear.^[^ ^7^, ^39^ ^]^ Given that MT_3_R activation during sleep leads to reduced stellate neuron activity, we hypothesized that this reduction might lower protein kinase activity and synaptic protein phosphorylation, thereby promoting synaptic shrinkage.

**Figure 5:**
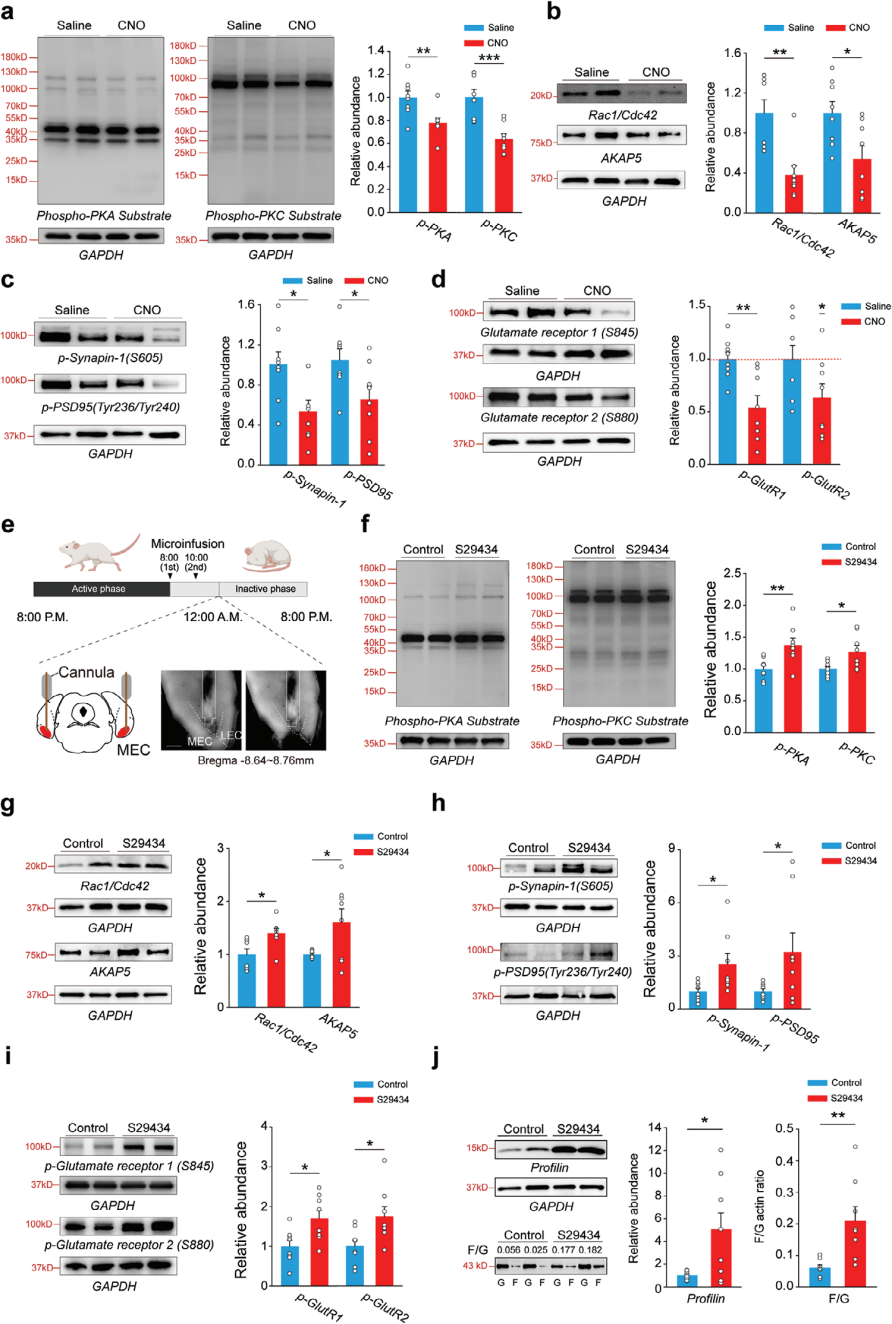
MT_3_R activation during sleep reduces protein kinase activity and dephosphorylates synaptic proteins. a) Immunoblot analysis of protein kinase phosphorylation levels after chemogenetic inhibition neurons in the superficial layer of the MEC during SD. p‐PKA (mean ± SEM), Student's t‐test, **P < 0.01, n = 8 rats for each group; p‐PKC (mean ± SEM), Student's t‐test, ***P < 0.001, n = 8 rats for each group. b) Immunoblot analysis of cytoskeleton co‐factors after chemogenetic inhibition neurons in the superficial layer of the MEC during SD. Rac1/Cdc42 (mean ± SEM), Welch's t‐test, **P < 0.01, n = 8 rats for each group; AKAP5 (mean ± SEM), Student's t‐test, *P < 0.05, n = 8 rats for each group. c) Immunoblot analysis of synaptic proteins phosphorylation levels after chemogenetic inhibition neurons in the superficial layer of the MEC during SD. p‐synapsin‐1 (mean ± SEM), Student's t‐test, *P < 0.05, n = 8 rats for each group; p‐PSD95 (mean ± SEM), Student's t‐test, *P < 0.05, n = 8 rats for each group. d) Immunoblot analysis of glutamate receptor subunit phosphorylation levels after chemogenetic inhibition neurons in the superficial layer of the MEC during SD. p‐GlutR1 (mean ± SEM), Student's t‐test, **P < 0.01, n = 8 rats for each group; p‐GlutR2 (mean ± SEM), One‐Sample t‐test, *P < 0.05, n = 8 rats for each group. e) The paradigm for superficial layers of the MEC collection after drug microinfusion during sleep. MEC, medial entorhinal cortex. Scale bar: 500 µm. f) Immunoblot analysis of protein kinase phosphorylation levels after administration of saline and S29434. p‐PKA (mean ± SEM), Student's t‐test, **P < 0.01, n = 8 rats for each group; p‐PKC (mean ± SEM), Welch's t‐test *P < 0.05, n = 8 rats for each group. g) Immunoblot analysis of cytoskeleton co‐factors after administration of saline and S29434. Rac1/Cdc42 (mean ± SEM), Student's t‐test, *P < 0.05, n = 8 rats for each group; AKAP5 (mean ± SEM), Student's t‐test, *P < 0.05, n = 8 rats for each group. h) Immunoblot analysis of synaptic protein phosphorylation levels after administration of saline and S29434. p‐synapsin‐1 (mean ± SEM), Student's t‐test, *P < 0.05, n = 8 rats for each group; p‐PSD95 (mean ± SEM), Mann‐Whitney Rank Sum Test, *P < 0.05, n = 8 rats for each group. i) Immunoblot analysis of glutamate receptor subunit phosphorylation levels after administration of saline and S29434. p‐GlutR1 (mean ± SEM), Student's t‐test, *P < 0.05, n = 8 rats for each group; p‐GlutR2 (mean ± SEM), Student's t‐test, *P < 0.05, n = 8 rats for each group. j) Immunoblot analysis of actin‐binding protein Profilin and cytoskeleton after administration of saline and S29434. profilin (mean ± SEM), Welch's t‐test, *P < 0.05, n = 8 rats for each group; F/G actin ratio (mean ± SEM), Welch's t‐test, **P < 0.01, n = 8 rats for each group. To standardize, the mean of the relative expression abundance (target protein grayscale value/GAPDH grayscale value) of each group's control was set as 1. p‐PKA, phosphorylation of Protein Kinase A; p‐PKC, phosphorylation of Protein Kinase C; AKAP5, A‐Kinase Anchor Protein 5; p‐PSD95, phosphorylation of Postsynaptic Density protein‐95.

To test this hypothesis, we assessed changes in protein kinase activity and synaptic protein phosphorylation in the MEC's superficial layers following chemogenetic inhibition of stellate neurons. We observed that inhibiting stellate neurons led to decreased phosphorylation levels of PKA and PKC, as well as reduced expression of Rac1 and A‐Kinase Anchor Protein 5 (AKAP5). Additionally, there was a significant reduction in the phosphorylation levels of key synaptic proteins, including synapsin‐1, postsynaptic density protein‐95 (PSD95), and the AMPAR subunits GluR1 and GluR2 in the MEC's superficial layers (**Figure** **5**a–d).

Next, we explored whether the melatonin/MT_3_R signaling pathway is responsible for these changes during sleep (Figure 5e). Blocking MT_3_Rs during sleep prevented the expected decrease in PKA and PKC activities and maintained high phosphorylation levels of synaptic proteins such as synapsin‐1, PSD95, GluR1, and GluR2 (Figure 5f–i), compared to control rats that experienced 4 h of sleep. This indicates that MT_3_R activation is crucial for the sleep‐associated downregulation of protein kinase activity and synaptic protein phosphorylation.

Moreover, the actin cytoskeleton, particularly filamentous actin (F‐actin), plays a vital role in maintaining synaptic structures, including dendritic spines. F‐actin is formed from globular actin monomers (G‐actin) under the regulation of actin‐binding proteins like Profilin, which facilitates actin polymerization.^[^ ^40^, ^41^ ^]^ Following MT_3_R blockade, we found that the F/G actin ratio and Profilin expression levels remained high and did not decrease during sleep (Figure 5j). In summary, our findings suggest that MT_3_R activation during sleep leads to reduced neural activity, which subsequently decreases protein kinase activity and synaptic protein phosphorylation. These molecular changes likely influence cytoskeletal dynamics, contributing to the shrinkage of dendritic spines in stellate neurons.

### 2.4 The Shrinkage of Dendritic Spines Mediated by MT_3_Rs is Associated with Spatial Memory Performance

Stellate neurons in the MEC are key players in spatial memory and path integration, projecting to the hippocampal dentate gyrus and CA3 regions, which are crucial for these functions.^[^ ^22^, ^42^ ^]^ Given that dendritic spine shrinkage during sleep might enhance signal‐to‐noise ratios, thus benefiting new memory acquisition upon waking,^[^ ^43^ ^]^ we investigated the link between MT_3_R‐induced dendritic spine shrinkage in stellate neurons and spatial memory performance.

**Figure 6:**
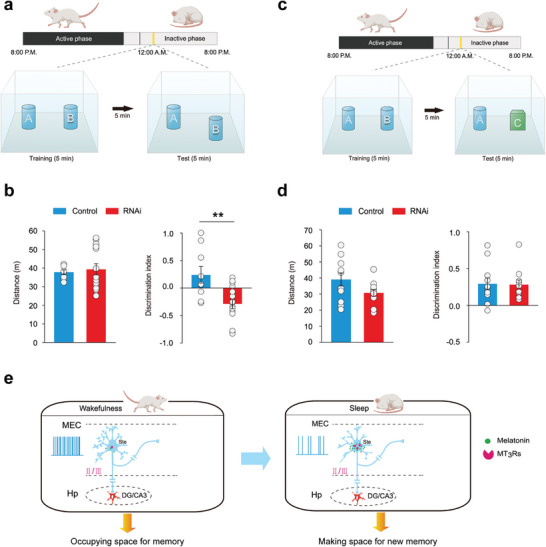
The shrinkage of dendritic spines mediated by MT_3_Rs is associated with the formation of spatial memory. a) Paradigms of behavior tests with the intervention of MT_3_Rs for exploring the role of MT_3_Rs in MEC‐dependent spatial memory acquisition. b) Knockdown of MT_3_Rs by RNAi during sleep in the MEC impaired spatial memory acquisition, right (mean ± SEM), Student's t‐test, **P < 0.01, n = 9 rats of control, n = 15 rats of RNAi, but did not affect the locomotion (mean ± SEM), left (mean ± SEM), Mann‐Whitney Rank Sum Test, *P* = 0.246, n = 8 rats of control, n = 14 rats of RNAi. c) Paradigms of behavior training and intervention of MT_3_Rs for exploring the role of MT_3_Rs in MEC‐independent object memory acquisition. d) Knockdown of MT_3_Rs by RNAi during sleep in the MEC did not affect the object memory acquisition, right (mean ± SEM), Mann‐Whitney Rank Sum Test, *P* = 0.849, n = 11 rats of control, n = 9 rats of RNAi, and the locomotion, left (mean ± SEM), Welch's t‐test, *P* = 0.141, n = 11 rats of control, n = 8 rats of RNAi. e) A model of the mechanism underlying the shrinkage of dendritic spines during sleep. During wakefulness, high activities of the stellate in the MEC related to memory acquisition would cause synaptic expansion. During sleep, melatonin/MT_3_R signaling could shrink dendritic spines and thus facilitates new memory formation post‐sleep. Ste, stellate neuron; Hp, hippocampus; DG, dentate gyrus.

We utilized an object‐place associative memory task to evaluate spatial memory in rats. Post MT_3_R gene knockdown, rats underwent new spatial learning after a 4 h sleep period (**Figure** **6**a). The results showed a significant decrease in the re‐exploration score of the displaced object. Similarly, blocking MT_3_Rs during sleep also led to a reduced re‐exploration score (Figure S12a,b, Supporting Information). This decrease was not due to changes in locomotor functions or exploratory motivation, as total running distance remained unaffected (Figure 6b). Furthermore, MT_3_R knockdown or blockade did not impact novel object memory acquisition (Figure 6c,d; Figure S12c,d, Supporting Information), consistent with previous findings that the MEC is more related to spatial rather than object memory.^[^ ^21^ ^]^

In another experiment, animals were trained in an object‐place associative memory task during the wake period, followed by MT_3_R blockade during the subsequent 4 h sleep period to assess effects on memory consolidation. The blockade of MT_3_Rs during sleep again decreased the re‐exploration score of the displaced object (Figure S13, Supporting Information). These findings suggest that MT_3_R‐mediated dendritic spine shrinkage during sleep is crucial for both the acquisition and consolidation of MEC‐related spatial memory (Figure 6e).

The positive impact of sleep‐induced dendritic spine shrinkage on memory formation indicates that SD might impair this essential process, leading to memory deficits. We tested whether chemogenetic inhibition of stellate neurons in the MEC could enhance dendritic spine shrinkage and mitigate memory impairments caused by SD. SD significantly hindered the rats' ability to recognize the altered spatial position of an object, reflected by a near‐zero discrimination index in the object‐place associative memory task. However, chemogenetic inhibition of stellate neurons during SD improved the discrimination index (Figure S14, Supporting Information), suggesting that this intervention can partially reverse the spatial memory deficits induced by SD.

## 3 Discussion

Sleep is known to induce synaptic structural changes, including dendritic spine shrinkage and modulation of AMPA‐type glutamate receptors, observed in cortical regions such as the primary motor and somatosensory cortex.^[^ ^7^, ^8^ ^]^ In this study, we provide compelling evidence of dendritic spine shrinkage during sleep specifically within the memory‐related MEC, where MT_3_R signaling plays a crucial role. Melatonin secretion from the pineal gland follows a circadian rhythm regulated by the light‐dark cycle, resulting in higher nighttime concentrations in the CSF and blood compared to daytime levels.^[^ ^11^, ^12^, ^13^, ^24^ ^]^ Extensive evidence suggests that besides the pineal gland, mitochondria in various tissues including the brain are significant sites of melatonin synthesis.^[^ ^44^, ^45^, ^46^ ^]^ AANAT, a key enzyme in melatonin synthesis, is highly expressed in the mitochondria of rat brain cells and hepatocytes.^[^ ^47^, ^48^ ^]^ Our findings indicate that the MEC shows high AANAT expression during the inactive phase and reduced expression during the active phase at night, suggesting that the MEC, similar to other neocortical regions, is capable of synthesizing melatonin. The rhythmic expression pattern of AANAT supports the notion that specific brain tissues exhibit distinct melatonin rhythms, with peak levels observed during the light period rather than the dark phase in rat cerebral cortex subcellular structures.^[^ ^29^ ^]^ Additionally, studies on MT_2_R knockout mice have shown reduced sleep specifically during the light phase, indicating that elevated melatonin levels during this period may influence sleep/wakefulness control regions in the brain.^[^ ^26^ ^]^ Using HPLC‐MS, our study directly measured higher melatonin levels in MEC brain tissue during the light period, providing evidence for an alternative source of melatonin secretion in rodents apart from the pineal gland, which may contribute to sleep‐related functions in rats during the light phase.

MT_3_Rs are considered putative intracellular receptors for melatonin,^[^ ^49^ ^]^ yet their physiological functions remain poorly understood. It is hypothesized that MT_3_R expression levels may exhibit circadian variation, peaking during sleep due to reduced acetylcholine and dopamine levels that disinhibit their expression.^[^ ^17^, ^50^ ^]^ Studies suggest that MT_3_Rs play a role in downregulating neuronal network excitability,^[^ ^17^, ^18^ ^]^ possibly influencing sleep‐related processes. These receptors are situated in proximity to mitochondria within cells, raising the possibility that they directly sense cytosolic melatonin, particularly in regions like the MEC where AANAT, a key enzyme in melatonin synthesis, shows elevated expression during sleep.^[^ ^51^, ^52^ ^]^ Unlike MT_1/2_Rs, MT_3_Rs exhibit distinct subcellular distribution and affinity profiles,^[^ ^53^, ^54^ ^]^ necessitating higher concentrations of exogenously delivered melatonin to activate them in experimental settings such as patch‐clamp studies. These findings suggest that extracellular melatonin may face challenges in accessing intracellular MT_3_Rs. Nevertheless, it remains plausible that melatonin synthesized in other brain regions during sleep could impact MEC function, underscoring the need for precise measurements of melatonin concentrations in stellate cells to elucidate intracellular melatonin sensing by MT_3_Rs.

Melatonin, primarily secreted by the pineal gland, enters the CSF and bloodstream, allowing it to exert diverse physiological functions including sleep promotion, antioxidant effects to mitigate oxidative stress, and regulation of the gonadal axis.^[^ ^14^, ^26^, ^53^ ^]^ During the light phase, melatonin facilitates sleep initiation primarily through G‐protein‐coupled MT_2_Rs.^[^ ^26^ ^]^ Previous studies have identified high expression of MT_1_Rs in various brain regions such as the retrosplenial cortex, dentate gyrus of the hippocampus, medial habenula, suprachiasmatic nucleus, superior colliculus, substantia nigra pars compacta, and dorsal raphe nucleus. MT_2_Rs, on the other hand, are predominantly observed in regions like the CA3 field of the hippocampus, reticular thalamic nucleus, supraoptic nucleus, inferior colliculus, substantia nigra pars reticulata, and ventrolateral periaqueductal gray.^[^ ^55^, ^56^ ^]^ Consistent with these reports, our findings reveal low expression of MT_1_Rs and MT_2_Rs in the MEC, contrasting with the high expression of MT_3_Rs. This suggests that MT_3_Rs in the MEC, particularly during sleep, may functionally interact with melatonin to mediate processes such as dendritic spine shrinkage. Understanding these distinct receptor effects enhances our grasp of melatonin's role across sleep phases and its broader implications in neural function.

During sleep, cellular structures, including dendritic spines mediated by MT_3_Rs, undergo shrinkage, potentially enhancing interstitial space and reducing synaptic weights. This phenomenon aids in clearing metabolic waste and conserving energy to maintain homeostasis.^[^ ^6^, ^43^, ^57^ ^]^ The expanded extracellular space during this process may facilitate the formation of new functional synapses and the enlargement of dendritic spines post‐sleep, thereby supporting the acquisition of new memories.^[^ ^5^ ^]^ Moreover, literature suggests that effective memory consolidation involves strengthening a select few synapses involved in memory encoding, while eliminating and shrinking synapses unrelated to memory is crucial. This selective process enhances the signal‐to‐noise ratio of memory‐encoded synapses.^[^ ^4^ ^]^

Our findings indicate that melatonin selectively promotes the shrinkage of immature dendritic spines, without significantly affecting the density of mushroom‐shaped spines crucial for memory. This selective effect suggests that melatonin may facilitate memory consolidation by primarily shrinking synapses unrelated to memory, thereby creating space for the expansion of memory‐related synapses. Further studies are warranted to elucidate these mechanisms comprehensively, including tracking dynamic changes in various dendritic spine types and investigating melatonin's effects post‐learning and memory acquisition.

The transition from wakefulness to sleep involves a series of neural and humoral regulatory changes. Wakefulness‐promoting systems, including monoaminergic and hypocretin peptidergic systems, experience reduced firing activity and transmitter release within the MEC, ultimately decreasing overall MEC excitability.^[^ ^21^, ^58^ ^]^ These changes likely create favorable conditions for dendritic spine shrinkage during sleep. Humoral factors such as adenosine, prostaglandin D_2_ (PGD_2_), and melatonin also play crucial roles during sleep with distinct kinetic profiles. Adenosine and PGD_2_ accumulate during wakefulness, acting as homeostatic regulators of sleep. Their concentrations peak during the transition from wakefulness to sleep and early sleep stages, rapidly declining thereafter. In contrast, melatonin secretion increases during sleep and persists for several hours, potentially exerting a sustained influence.^[^ ^59^ ^]^ Given our previous findings that adenosine inhibits MEC stellate neuron activity,^[^ ^60^ ^]^ it is plausible that adenosine initiates early dendritic spine shrinkage during sleep onset, while melatonin's sustained secretion may play a pivotal role in enduring dendritic spine dynamics.

Sleep promotes dendritic spine shrinkage in both adult male and female rats, a process dependent on MT_3_Rs, suggesting that melatonin‐mediated dendritic shrinkage is consistent across sexes. However, it is noteworthy that melatonin secretion declines with age.^[^ ^61^ ^]^ In our study, we observed impaired dendritic spine shrinkage during sleep in aged rats, accompanied by a significant reduction in dendritic spine density compared to adult rats. Given the essential role of melatonin/MT_3_R signaling in dendritic spine shrinkage during sleep, diminished melatonin levels associated with aging may hinder this process, leading to disrupted synaptic homeostasis. Consequently, the loss of dendritic spines and disruption of synaptic homeostasis could potentially contribute to age‐related cognitive impairment. Thus, our findings highlight MT_3_Rs as a promising target for addressing memory dysfunction in aging.

Despite differences in sleep rhythms between rats and humans, the fundamental functions of melatonin appear evolutionarily conserved. Melatonin's roles in promoting sleep during the light phase and reducing oxidative stress have been observed across nocturnal rodents and diurnal animals, including humans.^[^ ^26^, ^62^, ^63^, ^64^, ^65^ ^]^ Furthermore, numerous studies in humans have demonstrated that melatonin administration improves sleep disorders via MT_1/2_Rs and enhances memory and cognitive function.^[^ ^63^ ^]^ These insights suggest that MT_3_Rs may also contribute to dendritic spine shrinkage during sleep in diurnal animals like humans. However, further experiments are necessary to confirm this phenomenon in primates and other diurnal species.

In this study, we effectively prevented dendritic spine shrinkage by blocking MT_3_Rs using Golgi staining, a classical technique widely employed to detect dendritic spines' size and type changes during neural development, learning, and memory.^[^ ^66^, ^67^ ^]^ Notably, Golgi staining enabled the detection of MT_3_Rs‐mediated dendritic spine shrinkage during sleep without the need for additional techniques like two‐photon imaging, emphasizing its utility in studying static changes in dendritic morphology.

The mechanisms underlying MT_3_R‐mediated dendritic spine shrinkage likely involve multiple processes, including the downregulation of stellate neuron activity through potassium channel opening, reduction in PKA and PKC activity, and modulation of synaptic protein phosphorylation levels. This study encompasses various molecular processes, necessitating further exploration to elucidate the causal relationships between MT_3_R‐mediated changes in potassium channel opening, protein kinase activity, synaptic protein phosphorylation during sleep, and their impact on dendritic spine shrinkage. Furthermore, while our findings suggest an association between MT_3_Rs‐mediated dendritic spine shrinkage during sleep and spatial memory performance, establishing the causal relationship between dendritic spine shrinkage, post‐sleep memory acquisition, and memory consolidation requires further verification.

## 4 Conclusion

In conclusion, our study demonstrates that the homeostatic shrinkage of dendritic spines during sleep is dependent on melatonin acting through MT_3_Rs, revealing a novel molecular mechanism underlying sleep‐induced synaptic homeostasis. Traditionally, melatonin has been recognized for its role in sleep initiation through activation of G‐protein‐coupled MT_2_Rs. However, our findings highlight a distinct role for melatonin in mediating sleep‐related functions post‐sleep onset via intracellular MT_3_Rs. These results significantly broaden our comprehension of melatonin's contributions during the sleep phase.

## 5 Experimental Section

### Animals

All animal care and experimental procedures were conducted in strict accordance with the Army Medical University Guide for the Care and Use of Laboratory Animals (Approval number: SYXK‐PLA‐20120031). Unless otherwise specified, Sprague‐Dawley adult rats weighing between 300 and 400 g (8‐12 weeks) were utilized in this study. These rats were sourced from the Laboratory Animal Center at the Army Medical University and Beijing Vital River Laboratory Animal Technology Co., Ltd. The aging Sprague‐Dawley male rats (21 months old) were procured from Shulaibao (Wuhan) Biotechnology Co., Ltd. All experiment animals were housed under a 12 h light/12 h dark cycle, with lights on at 8:00 A.M., and maintained at a constant room temperature of 23 ± 1 °C, with ad libitum access to food and water. MT_3_R knockout rats were established by using CRISPR/Cas9 genome editing tools, in collaboration with Shanghai Biomodel Organism Science & Technology Development Co., Ltd (Shanghai, China). The main active site of the MT_3_R gene (exon4) was cleaved causing the nonsense‐mediated mRNA decay effect, resulting in the degradation of the target gene mRNA and the loss of gene function. The F0 generation rats obtained by microinjection of fertilized eggs were hybridized with each other to obtain heterozygous F1 generation rats. Then, the heterozygous F1 generation rats were mated with each other to obtain homozygous F2 generation animals for the experiment. To determine the genotypes of their offspring, the following primers were used to identify genotypes with polymerase chain reaction (PCR): 5′‐tccagaacatcccggctttatcc‐3′, 5′‐atctgctgggctggtgagttctat‐3′. To determine the expression of MT_3_R, the brain tissues containing the MEC were separated and evaluated by reverse transcription‐polymerase chain reaction (RT‐PCR) using the following primers: 5′‐agctctgaccagtgacatac‐3′, 5′‐ ccctatccatccagccttt‐3′.

### Stereotaxic Surgery

Rats were anesthetized with isoflurane and placed in a stereotaxic apparatus (RWD Life Technology Co, Ltd., Shenzhen, China). To prevent ocular dryness during the procedure, ophthalmic ointment or saline‐soaked cotton was used. The incision site was disinfected using iodine and medical alcohol. The scalp and periosteum were removed carefully to expose the skull surface. For drug application, the coordinates for the superficial layers of the MEC were −8.7 mm anteroposterior (AP), ±4.8 mm mediolateral (ML) and +4.3 mm dorsoventral (DV). Two small craniotomy holes were created, and then the cannulas were implanted and fixed firmly to the skull with the dental acrylic. For the surgical implantation of the electrode array,^[^ ^68^, ^69^ ^]^ a drug cannula containing an electrode array (The tip of the electrode array is 500 µm longer than that of the drug cannula) was implanted in the right superficial layers of the MEC. Under microscopic guidance, a bone window (1.5 mm × 1.5 mm) with a center at AP −8.7 mm and ML −4.8 mm (right side) was made for both electrode and guide cannula implantation. After removing the dura through a small burr hole, the drug cannula was lowered to the MEC (4.3 mm, vertical to the horizontal plane) with a speed of 1 µm s^−1^ controlled by an automatic micromanipulator (IVM‐1000, Scientifica). Once the electrode had reached the targeted superficial layers of the MEC with high signal‐to‐noise ratio spike activities, a drop of the agarose gel was carefully injected into the bone window to protect the brain tissue. Finally, the cannulas and electrodes were fixed to the skull using dental acrylic.

### RNAi

The RNAi experiment was conducted by injecting viruses into the superficial layer of MEC. Viruses for RNAi were generated using short hairpin RNA (shRNA) in collaboration with Shanghai Heyuan Biotechnology Co., Ltd. The pAAV‐U6‐shRNA‐CMV‐EGFP‐WPRE target gene ID is NM_0 010 0 4214.1, while the control virus pAAV‐U6‐NC‐CMV‐EGFP‐WPRE contains a random nonsense sequence. For viral injection, craniotomies were performed using stereotaxic coordinates (AP = −8.7 mm; ML = ± 4.8 mm; DV = + 4.3 mm). Bilateral injections of viruses (300 nL per injection site, titers > 10^12^) were administered slowly over 5 min using a glass cannula with a 10 µl syringe. Following a 3 min delay, the cannula was slowly removed. The incision was disinfected with 75% alcohol before being sutured with an absorbable suture. Three weeks after viral injection, morphological and behavioral experiments were conducted.

### Drug Application

S29434, purchased from Sigma–Aldrich (874484‐20‐5), was stored as stock solutions at −20 °C until use. Before administration, the drug stock solutions were freshly diluted to the required concentrations. At the time of injection (8:00 A.M. and 10:00 A.M.), 500 nL drug was injected into the MEC using a syringe pump (KD Scientific) at an infusion rate of 500 nL min^−1^. The injection cannula remained in place for 1 min after infusion. CNO (MedChemExpress, 34233‐69‐7) was dissolved in Dimethyl sulfoxide (DMSO, Sigma‐Aldrich, 67‐68‐5) and diluted in saline for a final concentration of DMSO of 0.4%.

### SD

Rats were subjected to SD for 4 h, commencing at 8:00 A.M. Briefly, rats were placed in a plastic cage containing novel nesting materials, food, and water, as well as climbing toys. When rats showed signs of sleep (reclining or lowering their heads), new climbing toys were introduced to keep them awake as described in our previous study.^[^ ^70^ ^]^ If these stimuli failed to arouse the animals' attention sufficiently, gentle tapping on the cage wall was employed to avoid any instances of falling asleep. The behavioral states of animals were monitored via video surveillance. Following a 4 h period of SD, brain tissue was extracted from rats at 12:00 A.M. for Golgi staining or immunoblotting.

### Actin Cytoskeleton Separation

Rats were deeply anesthetized with isoflurane and their brains were immediately immersed in the ice‐cold RNA solid Tissue RNA stable preservation solution (G3019, Servicebio). After 24 h of fixation and dehydration, brain slices measuring 300 µm in thickness were prepared using a vibratome (7000SMZ‐479, Campden Instruments). With the assistance of a stereomicroscope, 2–3 tissue blocks from the superficial layers of the MEC region were collected using a glass microtubule and preserved in ice‐cold RNAsolid Tissue RNA stable preservation solution.

### Sample Preparation of MEC tissue

Rats were anesthetized using isoflurane, and subsequently, their brains were immersed in ice‐cold artificial cerebrospinal fluid (ACSF) at 12:00 or 24:00. Brain slices measuring 300 µm in thickness were prepared using a vibratome. Under the guidance of a stereomicroscope, the superficial layers of the MEC region were dissected into 0.1–0.2 g tissue blocks and collected with ice‐cold RIPA protein lysate buffer (Thermo Fisher, 89 900) beforehand added Protease and Phosphatase Inhibitor Cocktail (Thermo Fisher, 78 442). In the chemogenetic inhibition experiment section, after a recovery period of 3 weeks following the injection of the chemogenetic virus, the animals were randomly divided into control and chemogenetic inhibition groups. During SD from 8:00 to 12:00, animals in the chemogenetic inhibition group were intraperitoneally injected with CNO (1 mg kg^−1^) at 8:00 and 10:00, while animals in the control group received intraperitoneal injections of saline. Then, two groups of rats were sampled at 12:00. In the experimental part of drug administration (S29434), after a recovery period of one week following the implantation of drug delivery cannulas in the superficial layers of the MEC, the animals were randomly divided into a treatment group and a control group. At 8:00 and 10:00, the treatment group received cannula administration of S29434 (50 µM, 0.5% DMSO in saline), while the control group was saline with 0.5% DMSO. After the administration, the animals were returned to home cages to be allowed for sleep freely. Subsequently, tissue samples were collected from both groups at 12:00. All samples were subsequently diluted with 5× SDS loading buffer (Beyotime, P0015) and subjected to immunoblotting further analysis.

### Immunoblotting Analysis

For analysis of the actin cytoskeleton, we followed the experimental standard outlined in the G‐Actin: F‐Actin In Vivo Assay Kit (BK037, Cytoskeleton). Tissue blocks were transferred into 1 mL Lysis and F‐actin stabilization buffer for lysis and homogenization via ultrasonication. After a 10 min lysis at 37 °C, the lysate was transferred to an ultracentrifuge tube and centrifuged at 100, 000 g (Beckman Coulter, Optima MAX‐XP) for 1 h to separate G‐actin (supernatant) and F‐actin (pellet). The pellets were subsequently resuspended in 1 mL of F‐actin depolymerization control solution and incubated on ice for 1 h. After dilution with 5× SDS loading buffer (Beyotime, P0015), the samples were analyzed by SDS–PAGE using mouse primary antibody anti‐Actin‐pan (Cytoskeleton, AAN02‐S). The band intensities were quantified using the Image Lab software. The F/G actin ratio was obtained by dividing the grayscale value of F‐actin by the grayscale value of G‐actin. Immunoblotting was performed following the standard protocol. Briefly, after ultrasonic crushing of the tissue block, the protein concentrations were determined by Bio‐Rad stain‐free gel for adjusting the loading volume to the same level. Antibody incubation and chemiluminescence were performed after protein transferring by semi‐dry method. The rabbit primary antibodies including anti‐AKAP5 (CST, 5671S), anti‐Phospho‐PSD95 (Tyr236/Tyr240) (CST, 3919S), anti‐Phospho‐Synapsin‐1(Ser605) (CST, 88246S), anti‐Rac1/Cdc42 (CST, 4651S), anti‐Phospho‐PKA Substrate (CST, 9624S), anti‐Phospho‐PKC Substrate Motif (CST, 6967S), anti‐GAPDH (HRP Conjugate) (CST, 8884S), anti‐Glutamate Receptor 1 (AMPA subtype) (phospho S845) (Abcam, ab76321), anti‐Ionotropic Glutamate receptor 2 (phospho S880) (Abcam, ab254321), anti‐Profilin (CST, 3237S), anti‐AANAT (FineTest, FNab00020), anti‐NQO2 (Abcam, ab181049), anti‐MTNR1A (Bioss, bs‐0027R), anti‐MTNR1B (Bioss, bs‐0963R), anti‐Vinculin (Proteintech, 26520‐1‐AP), and anti‐GAPDH (Beyotime, AF1186) were used. Bands were scanned and analyzed using an automated Image Lab. The indicated total protein amounts were expressed relative to GAPDH or Vinculin control signals.

### Golgi Staining and Dendritic Spine Detection

Golgi staining and dendritic spine counting were performed in accordance with our previous study.^[^ ^71^ ^]^ Briefly, rats were deeply anesthetized with isoflurane and their brains were quickly removed for Golgi staining using the Rapid Golgi Stain TM Kit (FD Neuro Technologies, Columbia, MD). The brains were first immersed in an impregnation solution composed of mercuric chloride, potassium dichromate, and potassium chromate for 14 days in the dark, followed by at least 3 days in a sucrose solution. Coronal sections (100 µm thick) from the MEC, embedded in 3.5% agarose gel, were obtained using a vibratome (VT1000, Leica Microsystems, Germany). The sections were mounted on adhesive glass slides and rinsed with distilled water before being immersed in a working solution. After dehydration and transparency treatment, they were finally sealed using a resinous medium. Stellate neurons located in layer II/III of the MEC were selected for inclusion in this study. For animals that received drug administration, only cells within 500 µm below the tip of the injection cannula were analyzed. For chemogenetic experiments, brain tissue was frozen and examined under a fluorescence microscope to confirm the accurate expression of the virus within the superficial layers of MEC. If the virus expression site is accurately identified, adjacent tissues were further used for Golgi staining and the cells within the viral expression regions were included in subsequent analyses. Dendritic spines were classified and quantified using a confocal microscope (LSM780/880/900, Zeiss, Jena, Germany) by an experimenter blinded to treatment conditions. Dendritic spines were quantified on second‐order dendrites of stellate neurons. Spine density was defined as the average number of spines per micron of dendrite length. The head diameter and length of each dendritic spine were measured using Zen 2012 software (Zeiss). There are four subtypes of dendritic spines; namely mushroom, stubby, thin, and branched. The classification criteria were established based on the previous study.^[^ ^71^ ^]^ Specifically, the head diameter of thin dendritic spines was smaller than the average length of their spine neck while their length exceeds 1 µm. The length of the stubby dendritic spines was less than 1 µm, with a length‐to‐head diameter ratio below 1. Mushroom dendritic spines had a head diameter twice the length of the spine neck, while branched dendritic spines possessed two or more heads.

### Multiple‐Channel Single‐Unit Recording

Multiple‐channel single‐unit recording in the superficial layers of the MEC was performed in accordance with our previous study.^[^ ^21^ ^]^ Following surgery, rats underwent a recovery period of at least one week, including 3–4 days of gentle acclimatization. After establishing a baseline recording for 60 min, drugs were slowly and unilaterally infused into the right MEC. An additional hour of recording immediately after drug infusion to continuously investigate the effects on neuronal activities in the MEC was conducted. The electrode array consisted of 12 insulated nichrome wires (30 µm in diameter, 100–250 kΩ, California Fine Wire), which were connected to a 16‐channel head stage equipped with a preamplifier (Digital Lynx SX; Neuralynx, MO) and the signals were acquired by Cheetah acquisition software (Cheetah 5.7.4). To acquire the spikes of neuron activity, a 16‐channel amplifier (Neuralynx) and band‐pass filtered at 300–10, 000 Hz was employed and amplified 10 000 times for each unit. All the LFP signals were digitized at a sampling rate of 30 kHz and band‐pass filtered (0.1–9 kHz) by an amplifier (Digital Lynx SX; Neuralynx, MO). The neural data recorded before and after drug infusion were merged using Matlab code to ensure that the analyzed effects on the same channel.

### Spike Sorting Analysis

SpikeSort3D 2.5.4 was used to analyze spike sorting. The KlustaKwik sorting method was employed to cluster waveforms exhibiting similar principal components. Based on consistent neuronal signal characteristics observed before and after drug infusion, it was determined that the same neuron was involved. Rate histograms were performed in NeuroExplorer Vision 5 to analyze the firing rates of neuron activity during Wake, NREM sleep, and REM sleep. To test the changes in firing rates after drug infusion, firing rates were normalized to baseline during the three stages separately.

### Whole‐Cell Patch Clamp Recording

Male Sprague‐Dawley rats (P14–20) were utilized for the preparation of acute brain slices following previously described methods in the previous study.^[34,^ ^72^ ^]^ Semi‐horizontal slices (400 µm) containing the superficial layers of entorhinal cortex were prepared with an oscillating tissue slicer (VT1000, Leica Microsystems, Germany) in an ice‐cold section solution (equilibrated with 95% O_2_ and 5% CO_2_, sucrose 220 mM; KCl 2.5 mM; NaH_2_PO_4_ 1.25 mM; NaHCO_3_ 26 mM; MgCl_2_ 6 mM; CaCl_2_ 1 mM; glucose, 10 mM). The slices were initially incubated for at least 40 min at room temperature (20–24 °C) in ACSF (NaCl 124 mM; KCl 3 mM; NaHCO_3_ 26 mM; MgCl_2_ 2 mM; CaCl_2_ 2 mM; glucose 10 mM) with oxygenation (95%O_2_–5%CO_2_). Then, the slices were transferred to a submerged chamber and continuously superfused with oxygenated (95% O_2_–5% CO_2_) ACSF at room temperature for recording. Neurons were visualized with an upright microscope with Leica differential interference contrast optics and an infrared video imaging camera. Glass pipettes (3–5 MΩ) filled with an internal solution (potassium gluconate 125 mM; KCl 20 mM; Hepes 10 mM; EGTA 1 mM; MgCl_2_ 2 mM; ATP 4 mM; adjusted to pH 7.2–7.4 with 1 M KOH) were used for whole‐cell recordings. Recording pipettes were positioned toward neurons of the slice under positive pressure. After the formation of the tight seal at least 1 GΩ made by negative pressure, the membrane patch was ruptured by suction. After stabilization for at least 5 mins, data were collected. Series resistance was compensated 50%–70% and cells were excluded from analyses if the series resistance increased by >15% during recording or exceeded 25 MΩ. Data were obtained with an EPC10 amplifier (HEKA Elektronik, Lambrecht/Pfalz, Germany) and analyzed with Pulse/Pulsefifit v.8.74 (HEKA Elektronik) and Igor Pro v.4.03 (WaveMatrics). To investigate the effects of melatonin, melatonin was puffed on the slice. Action potentials were determined automatically by using Mini‐analysis software (version 6.0, Synaptosoft). The PVT‐containing coronal brain slices (250 µm) were prepared from C3H/HeJ mice (6‐8 weeks) by using a vibroslicer (VT1000, Leica Microsystems) in ice‐cold ACSF. The ACSF had the following composition in mM: 119 NaCl, 2.5 KCl, 1.2 NaHPO_4_, 25 NaHCO_3_, 125 glucose, 2 MgCl_2_, and 2 CaCl_2_. It was continuously superfused with a mixture of 95% O_2_ and 5% CO_2_.

The slices were then incubated for 15 mins at a temperature of 32 °C in an incubation fluid with the following composition in mM: 110 NMDG, 2.5 KCl, 1.2 NaH₂PO₄, 25 NaHCO₃, 25 glucose, 10 MgSO₄, and 0.5 CaCl₂. This solution was adjusted to pH 7.3 with HCL and oxygenated with a mixture of 95%O₂ and 5%CO₂. Afterward, the slices were transferred to ACSF and continuously aerated with a mixture of 95% O₂ and 5% CO₂ at room temperature (20–24 °C) for at least 40 min before recording. During recording, the submerged slices were observed under an upright microscope equipped with Leica differential interference contrast optics and an infrared video imaging camera. Whole‐cell recordings were performed using patch electrodes pulled to tip resistances of 5–7 MΩ using a multistage puller (Sutter Instruments). These electrodes were filled with an internal solution having the following composition in mM: 125 potassium gluconate, 20 KCl, 10 HEPES, 1 EGTA, 2 MgCl_2_, 4 ATP, adjusted to pH 7.2–7.4 with KOH. Neurons were held at a membrane potential of −60 mV. After at least 5 min of stabilization, data were obtained using an EPC10 amplifier (HEKA Elektronik, Lambrecht/Pfalz, Germany), and conducted with Pulse/Pulsefit v8.74 (HEKA Elektronik) and Igor Pro v4.03 (WaveMatrics).

### scRT‐PCR

After whole cell recording, cellular cytoplasm was collected in an electrode tube containing 7 ul of the internal solution. The cDNA synthesis of each cell was performed using the PrimeScript II 1st Strand cDNA Synthesis Kit (6210A, TaKaRa) following the manufacturer's instructions as described previously. After denaturing at 65 °C for 5 min, the reaction mixture containing cytoplasm was cooled on ice and added to the reverse transcription system containing Oligo dT primer and random primers. The reaction was performed as follows: 60 min at 45 °C and 15 min at 70 °C. The resulting cDNA was utilized for detecting the presence of mRNAs coding for Gapdh, Calbindin, Reelin, and MT_3_R in the same cell. The first round of PCR amplification was performed with the gene‐specific multiplex primers using the SuperScript III One‐Step RT‐PCR Kit (12 574 026, Invitrogen). cDNA (1–2 ul) synthesized in the reverse transcription step was used as the template for the first round of PCR amplification, the reaction was carried out as follows: 2 min at 94 °C; 40 cycles of 15 s at 95 °C, 30 s at 55 °C, and 30 s at 68 °C; 5 min at 68 °C. Then PCR was performed with nested primers for each gene and the first PCR product (1–2 ul) was used as the template. The second reaction was carried out as follows: 2 min at 94 °C; 40 cycles of 15 s at 95 °C, 30 s at 55 °C, and 30 s at 68 °C, 5 min at 68 °C. Finally, the amplification products were visualized by electrophoresis in 2% agarose gels.

Primers (5′‐3′) and product sizes for single‐cell RT‐PCR: Gapdh (sense/anti‐sense): multiplex, 5′ ttaccagggctgccttctc/ acgccagtagactccacgac; nested, cttctcttgtgacaaagtggaca/ caccccatttgatgttagcg; Final product 200 bp; Calbindin (sense/anti‐sense): multiplex, 5′ gacgctgatggaagtggttac/ catccacggtcttgtttgct; nested, cgaaagaaggctggattgg/ agttgctggcatcgaaagag. Final product 152 bp; reelin (sense/anti‐sense): multiplex, 5′ tgggaacaacctcttcttcaatg/ cggaggcgtgtagaaccaga; nested, ctgggatgcggtaaaggtg/ attgctggagttgctgaagag. Final product 120 bp; MT_3_R (sense/anti‐sense): multiplex, 5′ aagcagggatgcacagtcac/ accggaaatctccattgacc; nested, atacaccatgaacttcgagcca/ cacagcaccctatccatccag. Final product 240 bp.

### Behavioral Tests

*Spatial memory acquisition test*: After knockdown of MT_3_R via RNAi, blockade of MT_3_R through drug administration (at 8:00 A.M and 10:00 A.M.) or chemogenetic inhibition of MT_3_R neuron in MEC(at 7:30 A.M and 10:00 A.M.), rats were subsequently allowed to freely explore two identical objects for 5 min in an experimental field measuring 80 by 80 cm, which was black and had cues such as a yellow square, purple pentagram, pink triangle, and orange circle on its four walls at the time of testing (12:00 A.M). Following a 5 min interval in their home cage, the rats underwent a test phase lasting 5 min. During the testing phase, object B's spatial position was altered, and rats were allowed to re‐explore both objects. Video recording was performed to document exploration and re‐exploration activities for further analysis.

*Spatial memory consolidation test*: The rats were given 20 min to freely explore two identical objects in an 80 by 80 cm experimental field at the time of acquisition (7:40 A.M.). The field was black and had cues, such as a yellow square, purple pentagram, pink triangle, and orange circle, on its four walls. After a 4 h interval in their home cage, the rats received MT_3_R‐blocking drugs at 8:00 A.M and 10:00 A.M. A 5 min test phase began at 12:00 A.M. During this phase, the position of object B was changed, and the rats had the opportunity to re‐explore both objects. Exploration and re‐exploration activities were recorded on video for further analysis.

*Object memory acquisition test*: Following knockdown of MT_3_R via RNAi or blockade of MT_3_R through drug administration (at 8:00 A.M and 10:00 A.M.), rats were subsequently allowed to freely explore two identical objects for 5 min in a black, 2D field at 12:00 A.M. After a 5 min interval in their home cage, the rats underwent a 5 min test phase during which they were permitted to re‐explore two objects; one object was replaced with a novel object. All behavioral experimental data were analyzed in a blinded manner. The discrimination index was calculated using the formula: (time spent at object B – time spent at object A)/(time spent at object B + time spent at object A).^[^ ^73^ ^]^ Only rats with immobilization time during exploration tasks less than < 80% were included for behavioral analyses, while animals with inaccurate cannula placement were excluded.

### Viral Injection and Chemogenetic Manipulations

Adeno‐associated virus (AAV2/9) vectors encoding a Cre‐dependent DREADD transgene, a mixture of rAAV2/9‐vglut2‐Cre‐WPRE‐hGH‐pA and rAAV2/9‐EF1a‐DIO‐hM4D‐mCherry, were purchased from Brain VTA (Wuhan) Co., Ltd. For virus injection, craniotomies were performed using stereotaxic coordinates (AP = −8.7 mm; ML = ±4.8 mm; DV = +4.3 mm). Bilateral injections of a virus mixture (300 nL per injection site, titers > 10^12^) were slowly administered over 5 min using a glass cannula and 10 µl syringe. After a delay of 3 mins, the cannula was carefully removed. The incision was disinfected with 75% alcohol and sutured with an absorbable suture.

At three weeks post‐virus injection, chemogenetic inhibition was induced through intraperitoneal (i.p.) administration of 1 mg kg^−1^ body weight of CNO.

### Electroencephalography (EEG)‐electromyogram (EMG) Recordings

The EEG‐EMG recordings were in accordance with previous studies.^[^ ^37^, ^74^ ^]^ To record EEG activity, it was carefully drilled two small holes in the frontal regions (coordinates: AP +2.0 mm, ML ± 2.0 mm) and one in the lateral parietal region (coordinates: AP −8.0 mm, ML +2.0 mm). Three tiny screws were drilled very carefully into the small holes near the cortex. Two EMG electrodes with leads were attached to the neck muscles and secured with sutures. All electrodes had been pre‐fixed to a micro‐pin connector. Rats were placed in a separate soundproofed recording room for recovery. The EEG‐EMG signals were amplified (Grass Link, Grass Technologies), band‐pass filtered (EEG: 0.3‐30 Hz, EMG: 10–100 Hz), and digitized at 128 Hz using sleep recording software (Vital Recorder, Kissei Comtec). By utilizing sleep analysis software (SleepSign for animals, Kissei Comtec), the polygraphic recording signals were initially automatically analyzed according to the spectral signatures of EEG‐EMG waveforms. Then, the stages of the sleep‐wake cycle were manually classified across consecutive non‐overlapping 4 s epochs. Wakefulness was identified by unsynchronized EEG rhythms with low amplitude and high frequency, accompanied by elevated EMG activity featuring phasic bursts. During NREM sleep, there was a synchronized and high‐amplitude EEG activity with low‐frequency (0.5–4 Hz), and lower EMG activity compared to arousal without phasic bursts. REM sleep was defined by the inclusion of a pronounced theta (4–10 Hz) rhythm and almost no EMG activity.

### Immunohistochemistry and Histological Identification

Adult Sprague‐Dawley rats were deeply anesthetized with isoflurane and perfused transcardially with 37 °C phosphate‐buffered saline (PBS), followed by fixation in 4% paraformaldehyde (PFA, 0–4 °C) in PBS. The brains were removed from the skull, postfixed in 4% PFA for an additional 12 h, and dehydrated with 30% sucrose at 4 °C for 24 h. Brain slices (40 µm) were prepared using a freezing microtome (CM 3050S, Leica). These slices were then stored in PBS at 4 °C until immunohistochemical processing. For immunohistochemical staining, slices were washed three times in 0.01 M PBS and then blocked with a solution containing 0.1% TritonX‐100 and 5% normal donkey serum (NDS; Jackson Immuno Research, PA, USA) in 0.01 M PBS at 37 °C for 30 min. Brain slices were subsequently incubated with primary antibodies in blocking solution at 4 °C for 24 h. Primary antibodies used were rabbit anti‐MT_3_Rs (Nqo2) (1:50, Affinity, DF7342), rabbit anti‐MT_1_Rs (1:200, Abbiotec, 250761), rabbit anti‐MT_2_Rs (1:200, Bioss, bs‐0963R), rabbit anti‐AANAT (1:200, Bioss, bs‐ 3914R), mouse anti‐Calbindin (1:100, Swant, 6B3), mouse anti‐reelin (CR‐50, 1:100, MBL, D223‐3) and rabbit anti‐c‐Fos (1:500, Abcam, ab190289) used. Sections were then washed three times in 0.01 M PBS (5 min each time) before being incubated with secondary antibodies in a blocking solution for 2 h at room temperature. For secondary antibody labeling, Alexa Fluor 488 donkey anti‐mouse was used (1:500; Thermo Fisher Scientific Technologies), 488 donkey anti‐rabbit, and 568 donkey anti‐rabbit (1:1000, Invitrogen) were used. Following washing with 0.01 M PBS, the slices were mounted on glass slides, air‐dried, and cover‐slipped using Fluoroshield with DAPI (Sigma–Aldrich). It was observed these sections with an LSM 900 confocal microscope (Carl Zeiss, Jena, Germany), and data analysis was conducted using Zen 2012 software (Zeiss). After conducting multiple‐channel single‐unit recordings, EEG‐EMG recordings, and behavioral tests, the rats were deeply anesthetized with isoflurane and transcardially perfused with 37 °C PBS followed by 4% PFA (0–4 °C). For the rats used in multiple‐channel single‐unit recording, an electrolytic lesion was created via a direct current of 0.01 mA applied for 10 s through the electrode before perfusion. The brains were extracted and immersed in a dehydration solution containing 30% sucrose and 4% paraformaldehyde. Continuous frozen sections of the MEC were collected and stained with DAPI to identify the recording or injection sites. Animals with incorrect recording or injection sites were excluded from subsequent analyses.

### FISH

The rats were anesthetized and underwent transcardial perfusion with 0.1 M PBS, followed by perfusion with 4% PFA in PBS. Subsequently, the brains were postfixed in 4% PFA at 4 °C for 24 h and then dehydrated using sucrose gradients (10%, 20%, and 30%). After freezing at −20 °C for at least 1 h in a freezing microtome, the brains were sectioned into coronal slices of thicknesses measuring 14 um. For the FISH, RNAscope Multiplex Fluorescent Assays V2 (Advanced Cell Diagnostics, ACD) were employed according to the manufacturer's instructions, utilizing probes specific to Nqo2 (514 231, ACD), Reelin (1 048 921, ACD), and Calbindin (417 551, ACD). Confocal microscopy (LSM780 or LSM880, Zeiss) was used to capture fluorescence images.

### Quantification of Melatonin Levels

*Preparation of MEC tissue samples*: After inducing deep anesthesia with isoflurane, the rats were immediately euthanized by cervical transection. Their brains were then extracted and placed in ACSF, and the MEC was dissected using a blade according to the rat brain localization Atlas (Charles Watson & George Paxinos, Fifth Edition). Following rinsing of the tissue surface with ACSF, excess water was absorbed using filter paper. The tissue blocks were weighed using an analytical balance and immediately cryopreserved in liquid nitrogen. Bilateral MEC tissues from rats were subsequently removed and homogenized with 1 ml of tissue homogenizer, supplemented with 500 µL 80% acetonitrile. The resulting homogenate was aspirated, and rinsed twice with 300 µL 80% acetonitrile on the tube walls and grinding bar, yielding ≈1 ml of tissue homogenate. After centrifugation at 7500 rpm for 20 min at 4 °C, the supernatant was obtained.

*Preparation of pineal gland tissue samples*: Following deep anesthesia with isoflurane, rats were euthanized by cervical dislocation, and their brains were extracted and placed in pre‐cooled artificial cerebrospinal fluid. Subsequently, the pineal gland was excised from the dorsal aspect of the third ventricle based on rat brain localization atlas. After rinsing with artificial cerebrospinal fluid (aCSF), excess water was blotted using filter paper and cryopreserved immediately in liquid nitrogen. The pineal gland tissue was extracted from – 80 °C, homogenized in 0.1 ml of tissue homogenizer with the addition of 100 µl 80% acetonitrile, thoroughly mixed, aspirated, and rinsed twice with 100 µl 80% to clean the tube wall, and grinding bar. A total volume of ≈300 µL of homogeneous tissue was obtained, which underwent centrifugation at 7500 rpm for 20 min at a temperature of 4 °C. The supernatant was then collected and evaporated until dryness.

*Collection of CSF samples*: After inducing deep anesthesia, the rats were secured onto an ex vivo positioning instrument with a flow rate of 2.0 L min^−1^ of 2% isoflurane to maintain anesthesia. The dorsal neck hair was removed using scissors and the skin was disinfected with iodophor before being clipped off with tissue scissors. The subcutaneous tissue and muscle were dissected to expose the cervical vertebrae, and a 1 ml syringe was inserted through the foramen magnum to obtain CSF). Careful attention was paid to ensure that the CSF obtained was clear and free of blood contamination. After completion of CSF extraction, the rats were euthanized by cervical transection, and the CSF was stored at – 80 °C. A 100 µL aliquot of rat CSF was mixed with 400 µL of 100% acetonitrile to achieve a final concentration of 80% acetonitrile, vortexed for 20 s, and sonicated for 20 min at 4 °C. The extracts were centrifuged at 7500 rpm for 20 mins at 4 °C, and the supernatants were collected and dried under vacuum at 45 °C.

### Quantification of Melatonin Levels

The melatonin concentration in the samples was quantified using a combination of HPLC‐MS (ExionLC and SCIEX Triple Quad 7500) under liquid‐phase conditions with mobile phase A consisting of 0.1% formic acid in water and mobile phase B consisting of 0.1% formic acid in acetonitrile at a flow rate of 0.3 ml/min, with an injection volume of 3 L. The Phenomenex bioZen Peptide XB‐C18 (50 × 2.1 mm 2.6 µm) chromatographic column was selected, and mass spectrometry conditions were set to positive ion mode. All experimental procedures were conducted under low light conditions to prevent melatonin photolysis.

### RNA Extraction, RT‐qPCR

The superficial layers of MEC samples were collected from rats 3 weeks after virus injection. Following deep anesthesia with isoflurane, rats were immediately sacrificed by cervical transection and their brains were rapidly dissected and submerged in ice‐cold RNAsolid Tissue RNA stable preservation solution (G3019, Servicebio). After 24 h of fixation and dehydration, a 300 µm brain slice was prepared using a vibratome. Under the guidance of a fluorescence microscope, the superficial layers of the MEC region expressing green fluorescence were dissected into 2–3 tissue blocks using a glass microtubule and preserved in ice‐cold RNAsolid Tissue RNA stable preservation solution. RNA extraction was performed using the phenol‐chloroform method with RNAiso Plus (TAKARA, 9109). The tissue blocks were lysed in 1 mL RNAiso Plus for 5 min, followed by the addition of 200 µL trichloromethane and intense shaking for 15 s. The mixture was then centrifuged at 12 000 rpm for 15 min at 4 °C. Isopropanol was added to the supernatant in a ratio of 1:1 and inverted twice, followed by incubation on ice for 10 min and subsequent centrifugation at 12 000 rpm for another 10 min at 4 °C. The supernatant was removed, followed by the addition of 75% ethanol and centrifugation at 7500 rpm for 10 mins at 4 °C. After removal of the supernatant again, RNA was dissolved in 20 µL of dd‐water and its concentration was measured using NanoDrop (Thermo Fisher).

Reverse transcription reaction was performed using PrimeScript RT Master Mix (TAKARA, RR036A). Subsequently, fluorescence quantitative PCR was performed using iTaq Universal SYBR Green (Bio‐Rad). The primer sequences used for quantitative PCR were as follows: Forword, 5′‐agctctgaccagtgacatac‐3′, Reverse, 5′‐ccctatccatccagccttt‐3′ (MT_3_R exon4 targeting); Forward, 5′‐agtggtgccagcaggttacg‐3′, Reverse, 5′‐aggtccgctccaacactatgc‐3′ (MT_1_R targeting); Forward, 5′‐ctgagtgtcattggctctgtcttc‐3′, Reverse, 5′‐gcaggctcggtggtaggtc‐3′(MT_2_R targeting); Forward, 5′‐cttctcttgtgacaaagtggaca‐3′, Reverse, 5′‐tgtccactttgtcacaagagaag‐3′ (GAPDH targeting).

Standard procedures were followed to obtain Ct values for MT_1_R, MT_2_R, MT_3_R and GAPDH in the quantitative PCR reaction. ΔΔ Ct was then calculated to reflect the abundance of MT_1_R, MT_2_R and MT_3_R RNA expression.

### Statistical Analysis

For immunoblotting analysis, the intensity ratio per target was calculated by dividing the raw intensity of each target by that of the corresponding GAPDH or Vinculin protein lane. To normalize the data for comparison between groups, these ratios were divided by the mean ratio in control groups. For behavioral experimental data analysis, the discrimination index was calculated using the formula: (time spent at object B – time spent at object A)/(time spent at object B + time spent at object A). All values were reported as means ± Standard Error of Mean (S.E.M.). Normality and homogeneity of variances were assessed for each group. For data with normal distribution and homogeneous variances, two‐tailed paired or unpaired *t*‐tests, analysis of variance (ANOVA) followed by Tukey‘s post hoc tests were conducted to compare estimates of variance between and within each of the sample groups. Welch *t*‐tests were employed for normally distributed data with non‐homogeneous variances. Non‐parametric tests including Mann‐Whitney rank sum test and Kruskal‐Wallis were used for non‐normally distributed data with non‐homogeneous variances. Statistical significance was set at *P < 0.05, **P < 0.01 and ***P < 0.001. The sample size, *P* value and the specific statistical test for each experiment were included in the figure legends. Statistical analyses were conducted utilizing Prism 8.0.1 (GraphPad, USA) and Sigmaplot 14.0 (Systat Software GmbH, Erkrath, Germany).

## Conflict of Interest

The authors declare no conflict of interest.

## Author Contributions

S.L. and X.L. contributed equally to this work. Z.A.H., C.H., S.C.R. and X.J.X. conceived the study. Z.A.H., C.H., X.J.X., H.G., and S.C.R. designed the experiments. S.Y.L., X.L., M.M.L., Q.H.C., D.Y., X.Q.Y., Z.L., N.W., J.H.J., Y.L.W., X.D.C., Y.X.L., F.L.L., J.Y. and C.H. executed the experiments and conducted statistical analysis. C.H., S.C.R. X.J.X. and Z.A.H. wrote the paper with the help of M.X., W.P.G., C.G.J., F.G.Y., D.G., X.D.T., Y.J.W., X.W.C. All authors read and commented on the manuscript.

## Supporting information

Supporting Information

## Data Availability

The data that support the findings of this study are available from the corresponding author upon reasonable request.
