# Supplementary material for: Homeostatic Shrinkage of Dendritic Spines Requires Melatonin Type 3 Receptor Activation During Sleep
Source: Adv Sci (Weinh). 2024 Aug 9;11(38):2400253. doi: 10.1002/advs.202400253 (PMC11481193; doi:10.1002/advs.202400253)
Supplement: Supplementary file 1 — Supporting Information [file ADVS-11-2400253-s001.docx]

Supporting Information

Homeostatic Shrinkage of Dendritic Spines Requires Melatonin Type 3 Receptor Activation During Sleep

Shiyin Li^1†^, Xin Li^1, 2†^, Minmin Lu^1^, Quanhui Chen^1^, Di Yao^3, 4^, Xiaoqian Yu^4^, Zhen Li^4^, Wuping Ge^4^, Na Wang^1^, Jiehua Jin^1^, Yaling Wang^1^, Yixiang Liao^1^, Fenlan Luo^1^, Jie Yan^1^, Xuedan Chen^5^, Chenggang Jiang^6^, Faguo Yue^7^, Dong Gao^8^, Xiangdong Tang^9^, Hong Guo^5^, Yanjiang Wang^10, 13^, Xiaowei Chen^11, 13^, Jianxia Xia^1^, Min Xu^12^, Shuancheng Ren^1*^, Chao He^1*^, Zhian Hu^1, 13*^

^†^These authors contributed equally.

^*^Corresponding authors: shuan0808@163.com (Shuancheng Ren); hechaochongqing@163.com (Chao He); zhianhu@aliyun.com ( Zhian Hu, lead contact).

This file includes:

Figures S1 to S14.


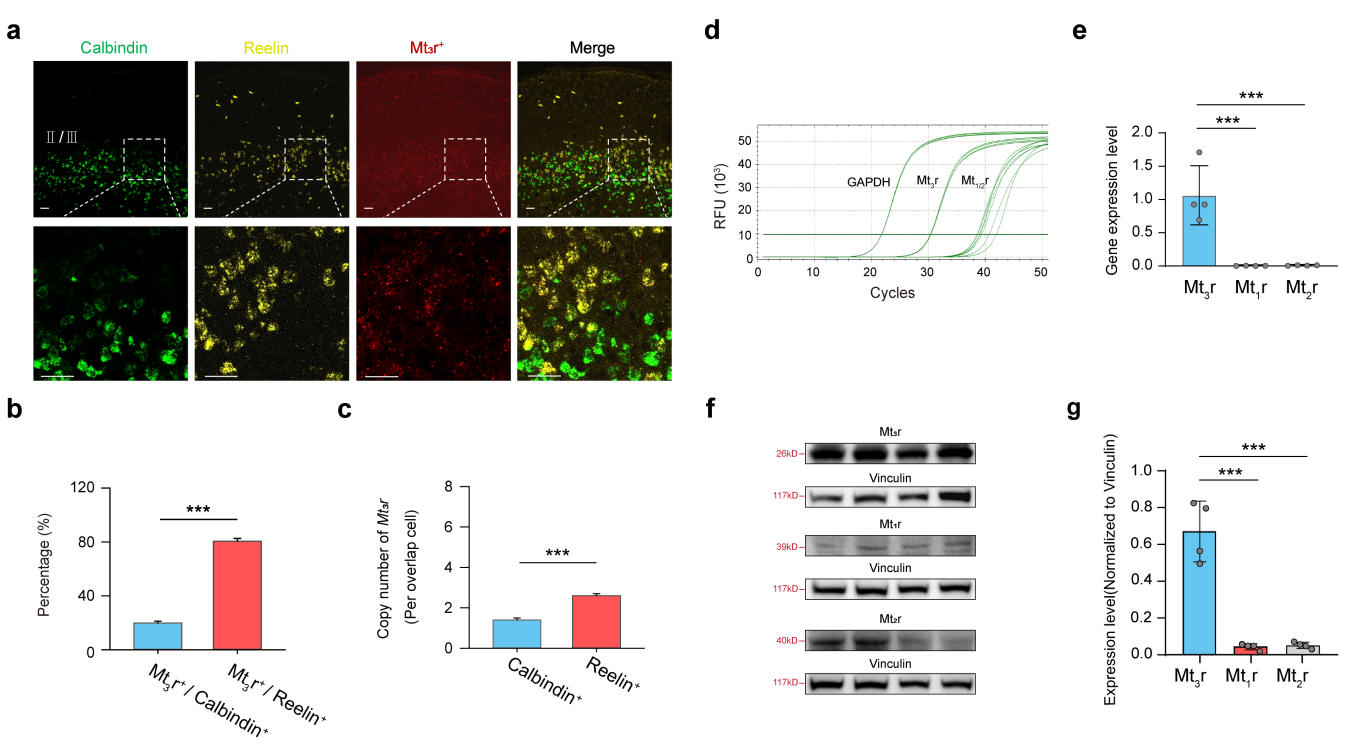


**Figure S1.** Selective expression of MT_3_Rs in MEC stellate neurons. a) Represented images of fluorescence in situ hybridization (FISH) showing the expression of MT_3_Rs in the reelin^+^ stellate neurons and calbindin^+^ pyramidal neurons. Scale bar: 50 μm. b) Percentage of reelin^+^ stellate neurons and calbindin^+^ pyramidal neurons that were positive for MT_3_Rs, noting that nearly 80% of the reelin^+^ stellate neurons were positive for MT_3_Rs (mean ± SEM). Student’s t-test, ****P* < 0.001, n = 20 sections from 4 rats. c) Copy number of MT_3_Rs in reelin^+^ stellate neurons and calbindin^+^ pyramidal neurons that were positive for MT_3_Rs (mean ± SEM). Mann-Whitney Rank Sum Test, ****P* < 0.001, n = 20 sections from 4 rats. d, e) The expression levels of MT_1_Rs, MT_2_Rs, and MT_3_Rs were determined using RT-qPCR (mean ± SEM). Kruskal-Wallis One Way Analysis of Variance on Ranks, ****P* < 0.001, n = 4 rats.

f, g) Immunoblot analysis of expression levels of MT_1_Rs, MT_2_Rs, and MT_3_Rs in MEC (mean ± SEM). Kruskal-Wallis One Way Analysis of Variance on Ranks, ****P* < 0.001, n = 4 rats.


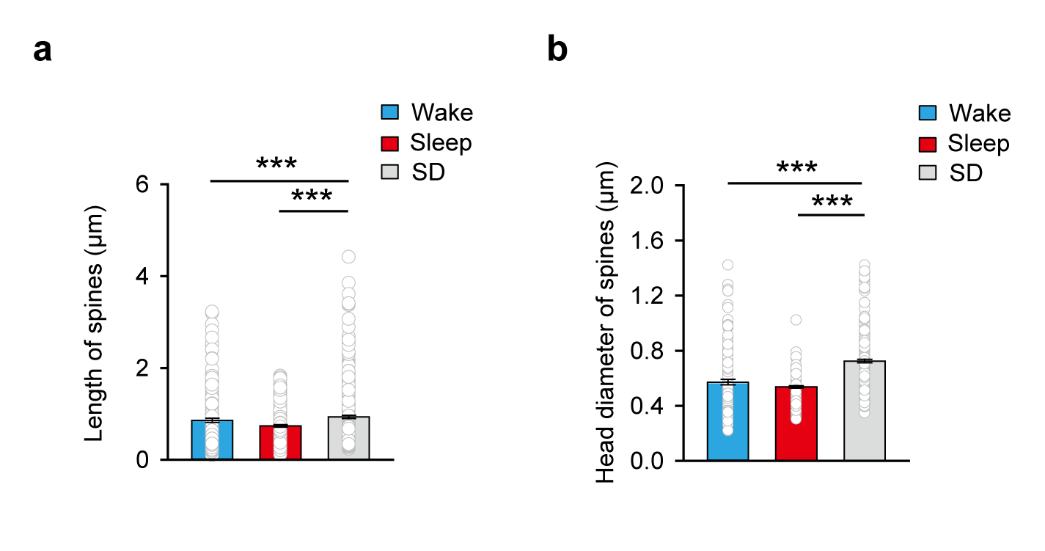


**Figure S2.** Sleep reduces the head diameter and length of dendritic spines in MEC stellate neurons. a, b) The head diameter (a) and length (b) of the dendritic spine in MEC stellate neurons after wake, sleep, and SD. Length (mean ± SEM), Kruskal-Wallis One Way Analysis of Variance on Ranks, ****P* < 0.001, n = 206 spines of wake, n = 208 spines of sleep, n = 333 spines of SD; Head diameter (mean ± SEM), Kruskal-Wallis One Way Analysis of Variance on Ranks, ****P* < 0.001, n = 157 spines of wake, n = 143 spines of sleep, n = 274 spines of SD, wake vs sleep.


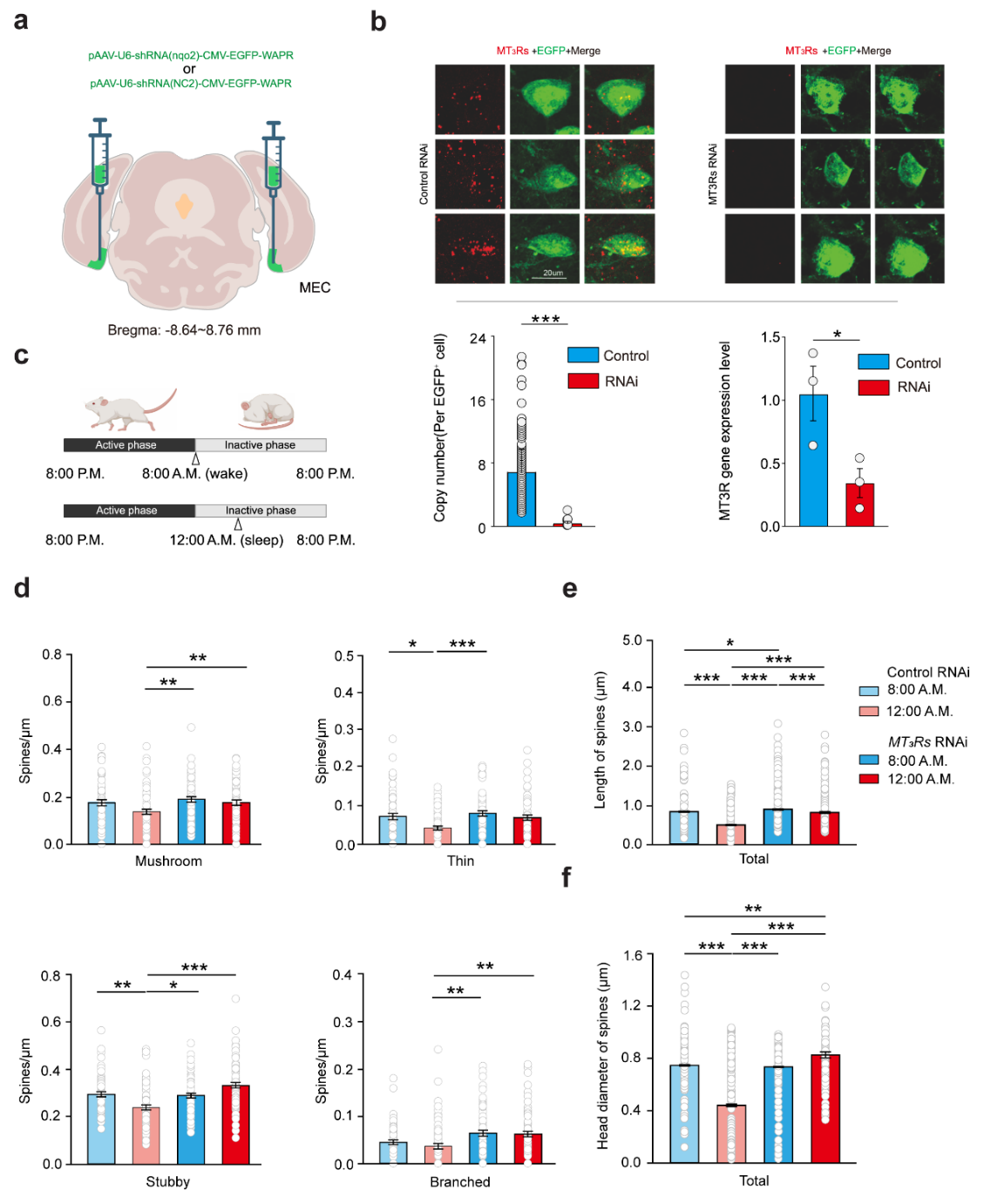


**Figure S3.** Knockdown of MT_3_Rs hinders the shrinkage of different types of dendritic spines in MEC stellate neurons during sleep. a) Schematic representation of MT_3_Rs RNA interference (RNAi)-directed transcript knockdown. b) FISH (mean ± SEM, Mann-Whitney Rank Sum Test, ****P* < 0.001, n = 116 cells for control, n = 126 cells for RNAi) and quantitative PCR reaction (mean ± SEM, Student's t-test, **P* < 0.05, n = 3 rats) showing the effects of RNAi on MT_3_R expression in MEC. c) Paradigm for detecting dendritic spines in MEC stellate neurons during sleep after knockdown of MT_3_Rs. d) Alterations of different subtype dendritic spine density of stellate neurons after knockdown of MT_3_Rs by RNAi. mushroom (mean ± SEM), Kruskal-Wallis One Way Analysis of Variance on Ranks, ***P* < 0.01, n = 57 cells of control 8:00 A.M., n = 69 cells of control 12:00 A.M., n = 65 cells of RNAi 8:00 A.M., n = 80 cells of RNAi 12:00 A.M.; thin (mean ± SEM), Kruskal-Wallis One Way Analysis of Variance on Ranks, **P* < 0.05, ****P* < 0.001, n = 57 cells of control 8:00 A.M., n = 69 cells of control 12:00 A.M., n = 65 cells of RNAi 8:00 A.M., n = 80 cells of RNAi 12:00 A.M.; stubby (mean ± SEM), Kruskal-Wallis One Way Analysis of Variance on Ranks, **P* < 0.05, ***P* < 0.01, ****P* < 0.001, n = 57 cells of control 8:00 A.M., n = 69 cells of control 12:00 A.M., n = 65 cells of RNAi 8:00 A.M., n = 80 cells of RNAi 12:00 A.M.; branched (mean ± SEM), Kruskal-Wallis One Way Analysis of Variance on Ranks, ***P* < 0.01, n = 57 cells of control 8:00 A.M., n = 69 cells of control 12:00 A.M., n = 65 cells of RNAi 8:00 A.M., n = 80 cells of RNAi 12:00 A.M. e, f) Changes in length (e) and head diameter (f) of dendritic spine in MEC stellate neurons after knockdown of MT_3_Rs. length (mean ± SEM), Kruskal-Wallis One Way Analysis of Variance on Ranks, **P* < 0.05, ****P* < 0.001, n = 1430 spines of control 8:00 A.M., n = 1064 spines of control 12:00 A.M., n = 1730 spines of RNAi 8:00 A.M., n = 2254 spines of RNAi 12:00 A.M.; head diameter (mean ± SEM), Kruskal-Wallis One Way Analysis of Variance on Ranks, ***P* < 0.01, ****P* < 0.001, n = 1130 spines of control 8:00 A.M., n = 877 spines of control 12:00 A.M., n = 1321 spines of RNAi 8:00 A.M., n = 1795 spines of RNAi 12:00 A.M..


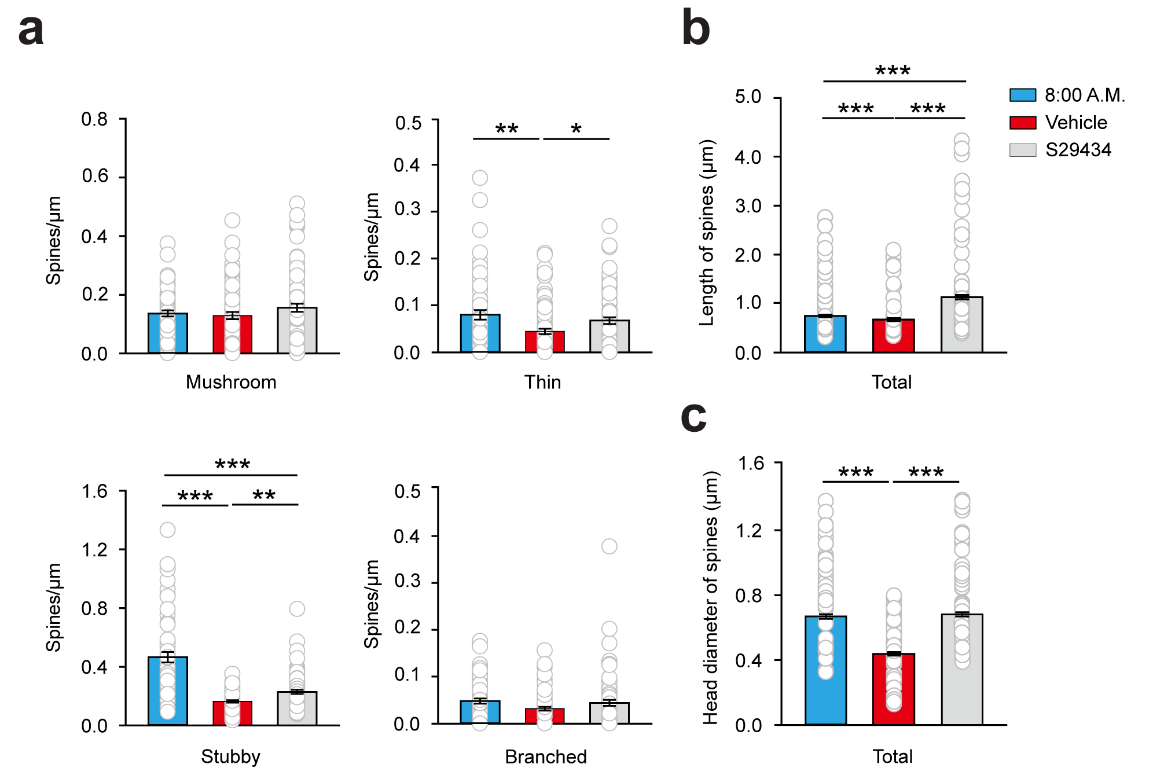


**Figure S4.** Blockade of MT_3_Rs during sleep hinders shrinkage of different types of dendritic spines**.** a) Alterations in the subtype dendritic spine density of stellate neurons after blockade of MT_3_Rs during sleep. mushroom (mean ± SEM), Kruskal-Wallis One Way Analysis of Variance on Ranks, *P* > 0.05, n = 60 cells of control 8:00 A.M., n = 80 cells of Vehicle, n = 79 cells of S29434; thin (mean ± SEM), Kruskal-Wallis One Way Analysis of Variance on Ranks, **P* < 0.05, ***P* < 0.01, n = 60 cells of control 8:00 A.M., n = 80 cells of Vehicle, n = 79 cells of S29434; stubby (mean ± SEM), Kruskal-Wallis One Way Analysis of Variance on Ranks, n = 60 cells of control 8:00 A.M., ***P* < 0.01, ****P* < 0.001, n = 80 cells of Vehicle, n = 79 cells of S29434; branched (mean ± SEM), Kruskal-Wallis One Way Analysis of Variance on Ranks, *P* > 0.05, n = 60 cells of control 8:00 A.M., n = 80 cells of Vehicle, n = 79 cells of S29434. b, c) Changes in the length (b) and head diameter (c) of the dendritic spine of stellate neurons after blockade of MT_3_Rs during sleep. length (mean ± SEM), Kruskal-Wallis One Way Analysis of Variance on Ranks, ****P* < 0.001, n = 287 spines of control 8:00 A.M., n = 252 spines of Vehicle, n = 346 spines of S29434; head diameter (mean ± SEM), Kruskal-Wallis One Way Analysis of Variance on Ranks, ****P* < 0.001, n = 245 spines of control 8:00 A.M., n = 181 spines of Vehicle, n = 246 spines of S29434.


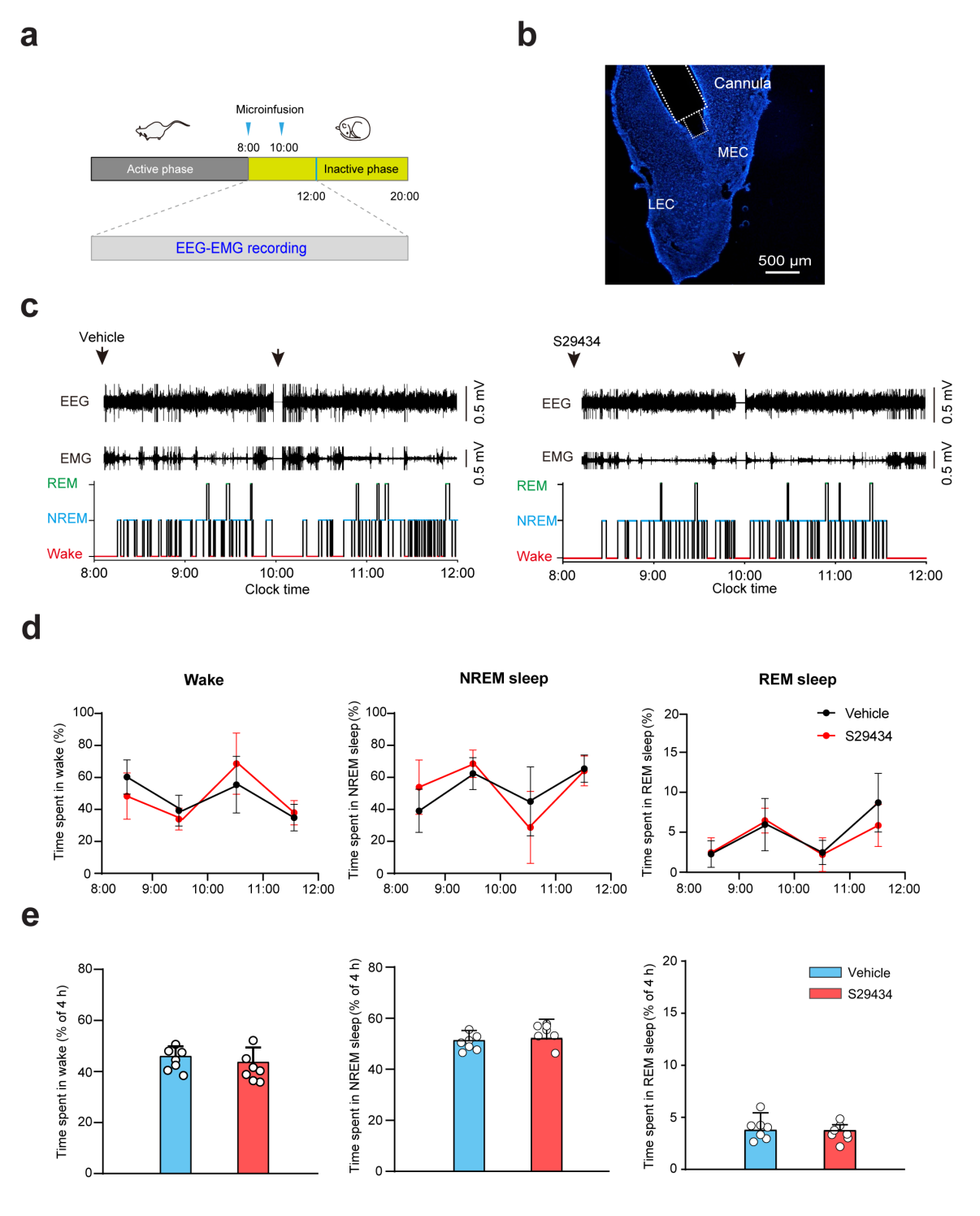


**Figure S5.** Inhibition of MT_3_Rs in the MEC does not affect sleep-wake behavior. a) Paradigm for exploring the effect of MT_3_Rs on sleep-wake behavior. b) A represented image showing the microinjection sites. c) Reprehensive electroencephalogram (EEG), electromyogram (EMG) traces, and hypnograms during 4 h after saline (left) and S29434 (right) injections. d, e) Quantitative analyses of time spent in NREM, wake, and REM states. Wake (mean ± SEM): Mann-Whitney Rank Sum Test, *P* =0.128, n = 7 rats for each group; NREM (mean ± SEM): Mann-Whitney Rank Sum Test, *P* =0.097, n = 7 rats for each group; REM (mean ± SEM): Student’s t-test, *P* = 0.695, n = 7 rats for each group.


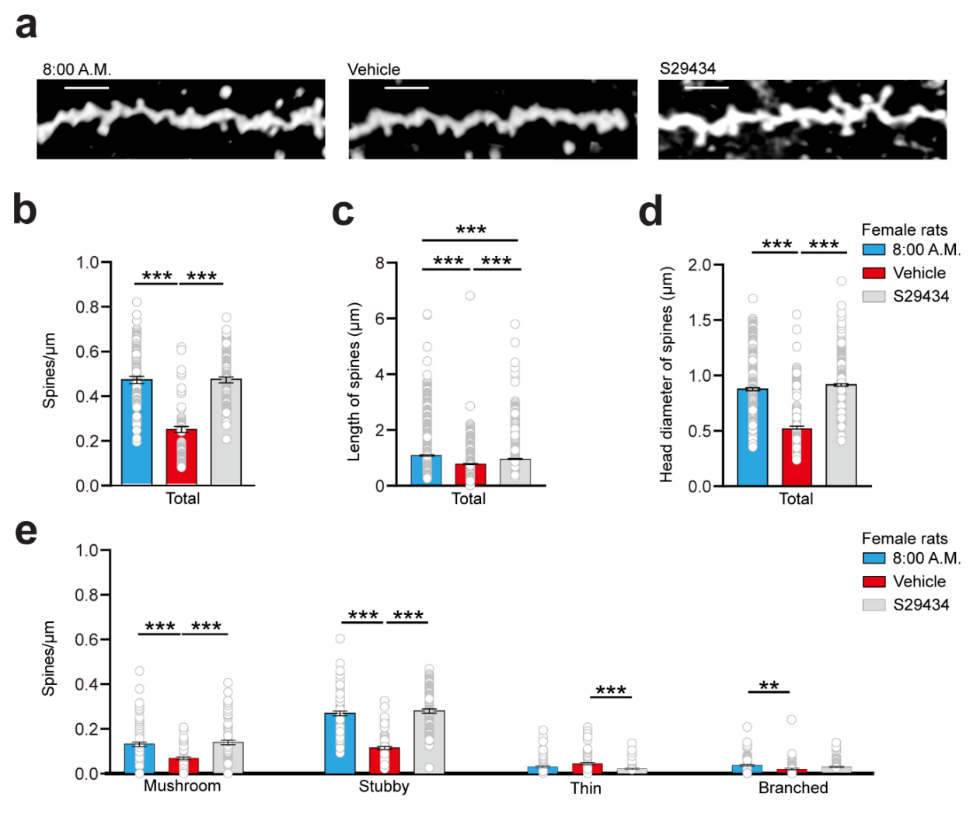


**Figure S6.** MT_3_R activation promotes the shrinkage of dendritic spines during sleep in female rats. a) Representative images of Golgi staining of dendritic spines in different groups. Scale bar: 10 μm. b) Changes in the dendritic spine density of the stellate neurons in female rats after blockade of MT_3_Rs by S29434 during sleep (mean ± SEM). Kruskal-Wallis One Way Analysis of Variance on Ranks, ****P* < 0.001, n = 80 cells of 8:00 A.M., n = 79 cells of vehicle, n = 80 cells of S29434. c, d) The length (c) and head diameter (d) of the dendritic spine in MEC stellate neurons in female rats after 8:00 A.M., vehicle and S29434. Length (mean ± SEM), Kruskal-Wallis One Way Analysis of Variance on Ranks, ****P* < 0.001, n = 847 spines of 8:00 A.M., n = 681 spines of vehicle, n = 885 spines of S29434; Head diameter (mean ± SEM), Kruskal-Wallis One Way Analysis of Variance on Ranks, ****P* < 0.001, n = 319 spines of 8:00 A.M., n = 207 spines of vehicle, n = 324 spines of S29434. e) Variation in the density of different subtypes of dendritic spines in stellate neurons was observed in female rats after blocking MT_3_Rs with S29434 during sleep. mushroom (mean ± SEM): Kruskal-Wallis One Way Analysis of Variance on Ranks, ****P* < 0.001, n = 80 cells of 8:00 A.M., n = 79 cells of vehicle, n = 80 cells of S29434; stubby (mean ± SEM): Kruskal-Wallis One Way Analysis of Variance on Ranks, ****P* < 0.001, n = 80 cells of 8:00 A.M., n = 79 cells of vehicle, n = 80 cells of S29434; thin (mean ± SEM): Kruskal-Wallis One Way Analysis of Variance on Ranks, ****P* < 0.001, n = 80 cells of 8:00 A.M., n = 79 cells of vehicle, n = 80 cells of S29434; branched (mean ± SEM): Kruskal-Wallis One Way Analysis of Variance on Ranks, ***P* < 0.01, n = 80 cells of 8:00 A.M., n = 79 cells of vehicle, n = 80 cells of S29434.


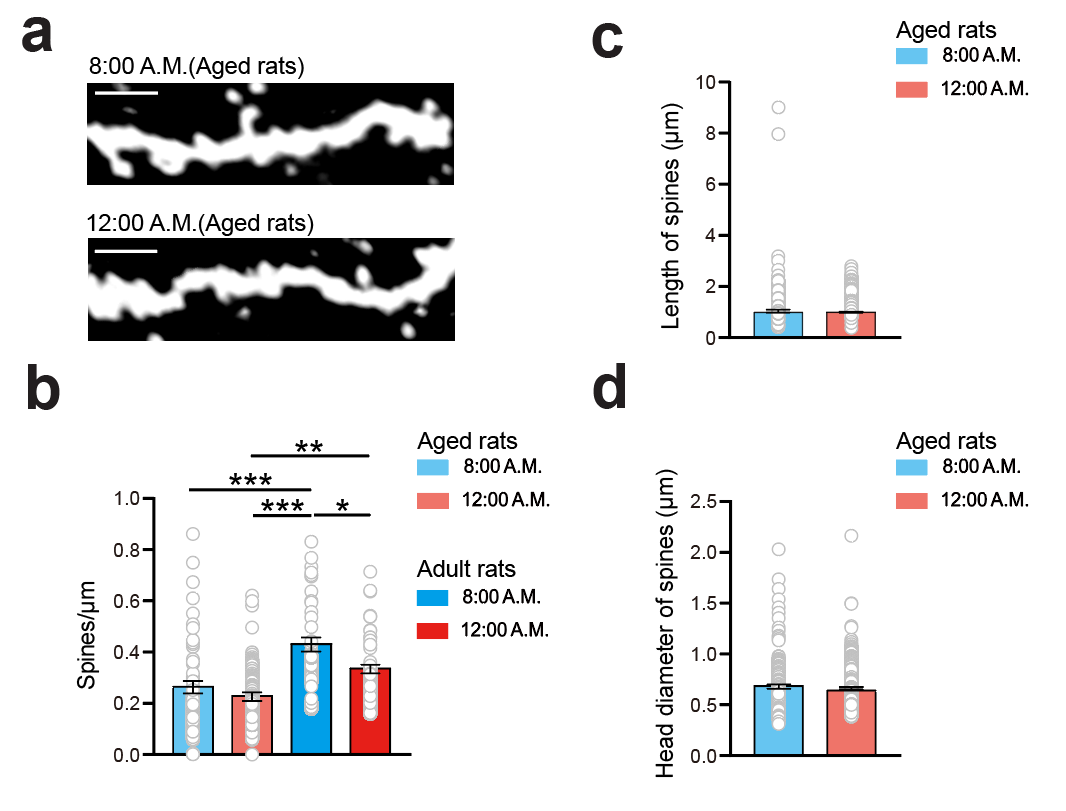


**Figure S7.** The morphological shrinkage of dendritic spines during sleep was not obvious in aged rats. a) Schematic representation of Golgi staining of dendritic spines in aged rats. Scale bar: 10 μm. b) Alterations of dendritic spine density of stellate neurons in aged and adult rats (mean ± SEM). Kruskal-Wallis One Way Analysis of Variance on Ranks, **P* < 0.05, ***P* < 0.01, ****P* < 0.001, n = 63 cells of aged rats 8:00 A.M., n = 65 cells of aged rats 12:00 A.M., n = 45 cells of adult rats 8:00 A.M., n = 56 cells of adult rats 12:00 A.M.. c, d) The length (c) and head diameter (d) of the dendritic spine in MEC stellate neurons in aged rats. length (mean ± SEM), Unpaired t-test, *P* = 0.920, n = 226 spines of aged rats 8:00 A.M., n = 556 spines of aged rats 12:00 A.M. head diameter (mean ± SEM), Unpaired t-test, *P* =0.5205, n = 160 spines of aged rats 8:00 A.M., n = 312 spines of aged rats 12:00 A.M.


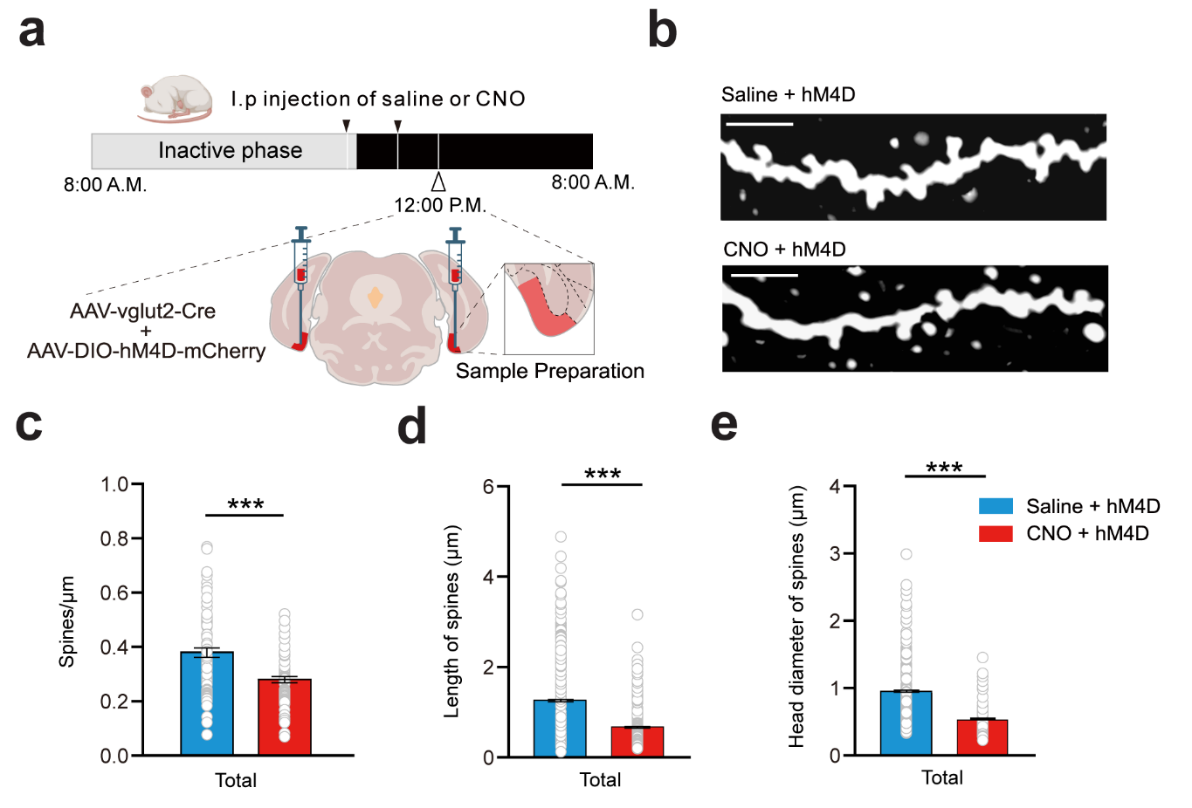


**Figure S8.** Downregulation of stellate neuron activity induces shrinkage of dendritic spines during the active phase. a) Schematic diagram for detecting dendritic spines after selective inhibition of MT_3_R^+^ stellate neurons during wakefulness. b) Representative images of Golgi staining of dendritic spines in different groups. Scale bar: 10 μm. c) Changes in the dendritic spine density of MT3R^+^ stellate neurons after chemogenetic inhibition (mean ± SEM). Unpaired t-test, ****P* < 0.001, n = 78 cells of saline, n = 79 cells of CNO d, e) The length (d) and head diameter (e) of the dendritic spine in MEC stellate neurons after inhibition of the stellate neuron activity. length (mean ± SEM), Unpaired t-test, ****P* < 0.001, n = 865 spines of saline, n = 369 spines of CNO; head diameter (mean ± SEM), Unpaired t-test, ****P* < 0.001, n = 563 spines of saline, n = 303 spines of CNO.


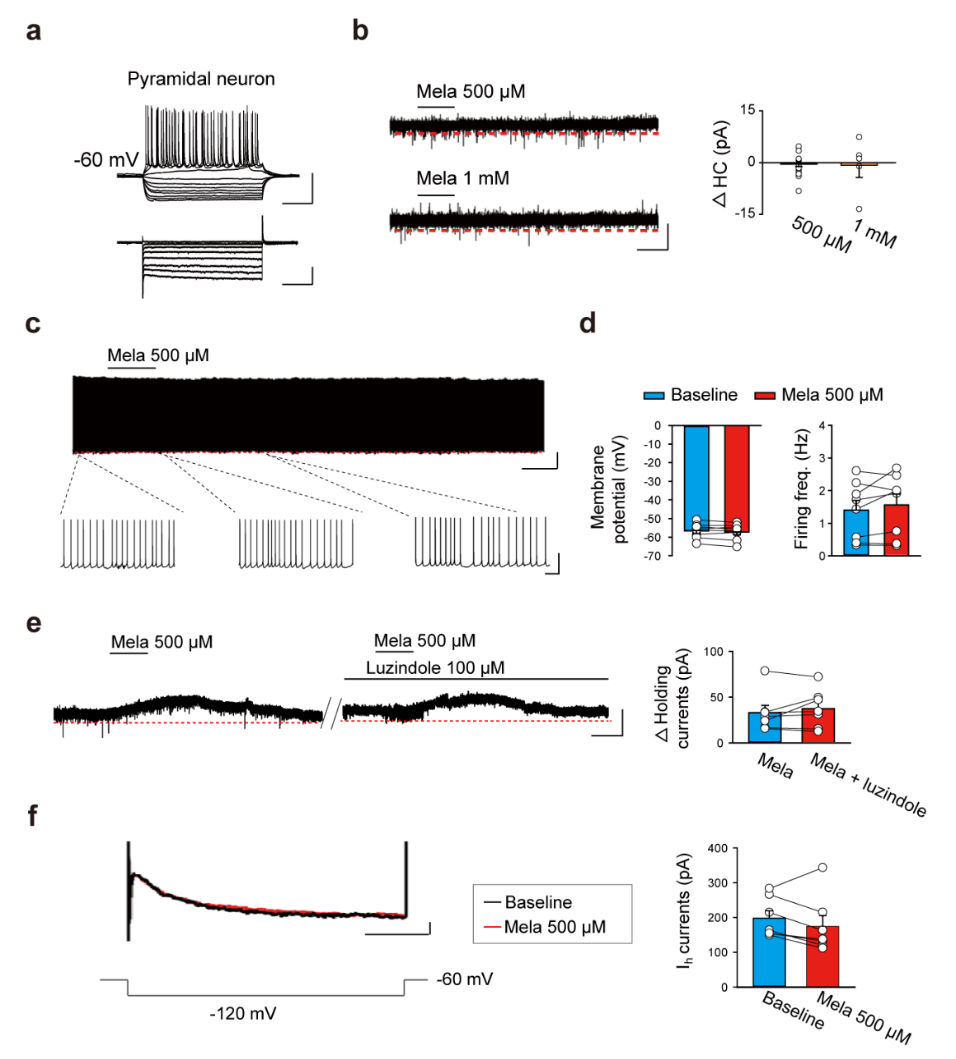


**Figure S9.** MT_3_R activation downregulates the activity of stellate neurons *in vitro*. a) Electrophysiological properties of a pyramidal neuron. Top, Scale bar: 250 ms, 50 mV. Down, scale bar: 250 ms, 100 pA. b) Effects of melatonin on the holding currents in the pyramidal neurons. Scale bar: 100 S, 40 pA . 500 uM (mean ± SEM), Paired t-test, *P* = 0.596, n = 19 cells; 1mM (mean ± SEM), Paired t-test, *P* = 0.828, n = 5 cells. c,d) Melatonin did not affect the membrane potentials and firing frequency in the pyramidal neurons. Top, scale bar: 100 S, 20 mV. Down, scale bar: 1 S, 40 mV. Membrane potential (mean ± SEM): Paired t-test, *P =* 0.135, n = 8 cells; Firing freq (mean ± SEM): Paired t-test, *P =* 0.227, n = 8 cells. e) Melatonin-induced outward currents in stellate neurons were not affected by MT_1/2_R antagonists (mean ± SEM). Scale bar: 100 S, 40 pA. Paired t-test, *P =* 0.318, n = 7 cells. f) Melatonin did not affect the I_h_ currents (mean ± SEM). Scale bar: 250 ms, 100 pA. Wilcoxon Signed Rank Test, *P* = 0.297, n = 7 cells.


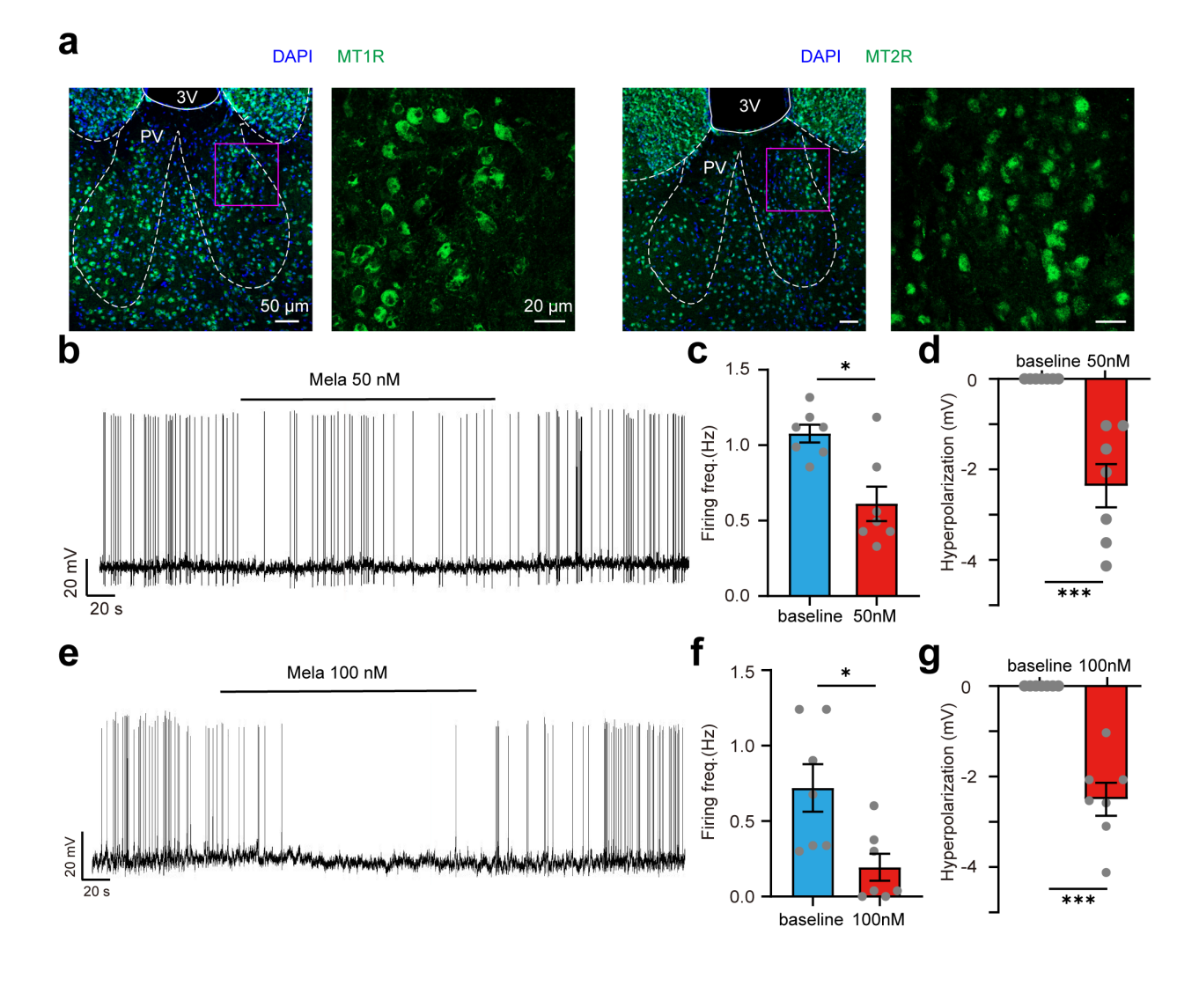


**Figure S10.** Melatonin inhibits the activity of the paraventricular nucleus of the thalamus (PVT) neurons**.** a) Distribution of different melatonin receptors in the PVT brain region. b to d) Effects of melatonin (50 nM) on the membrane potentials and firing rates of PVT neurons. firing rate (mean ± SEM): Wilcoxon Signed Rank Test, **P* < 0.05, n = 7 cells; hyperpolarization (mean ± SEM): Paired t-test, ****P* < 0.001, n = 7 cells. e to g) Effects of melatonin (100 nM) on the membrane potentials and firing rates of PVT neurons. firing rate (mean ± SEM): Wilcoxon Signed Rank Test, **P* < 0.05, n = 7 cells. hyperpolarization(mean ± SEM): Paired t-test, ****P* < 0.001, n = 7 cells.


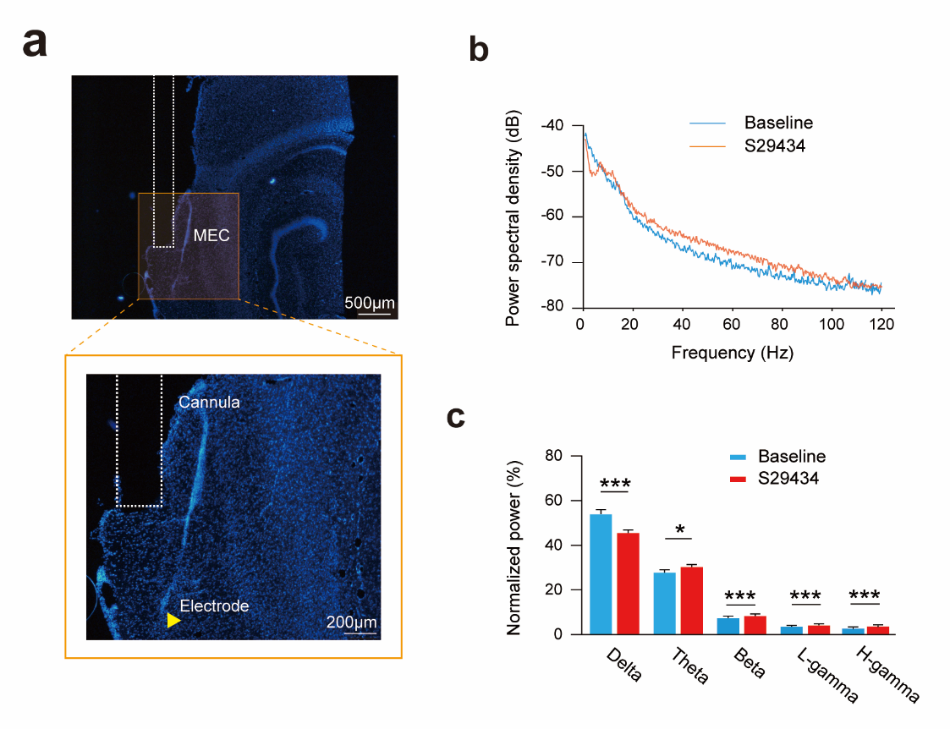


**Figure S11.** MT_3_R activation downregulates neural activity during sleep. a) An image showing the electrode and cannula implanted in the layer II of the MEC. b, c) Blocking MT_3_Rs decreased the delta oscillation, but increased other frequency band oscillations in the superficial layers of MEC. Delta (mean ± SEM): Wilcoxon Signed Rank Test, ****P* < 0.001, n = 52 channels from 6 rats; Theta (mean ± SEM): Paired t-test, **P* < 0.05, n = 52 channels from 6 rats; Beta (mean ± SEM): Paired t-test, ****P* < 0.001, n = 52 channels from 6 rats; L-gamma (mean ± SEM): Wilcoxon Signed Rank Test, ****P* < 0.001, n = 52 channels from 6 rats; H-gamma (mean ± SEM): Wilcoxon Signed Rank Test, ****P* < 0.001, n = 52 channels from 6 rats.


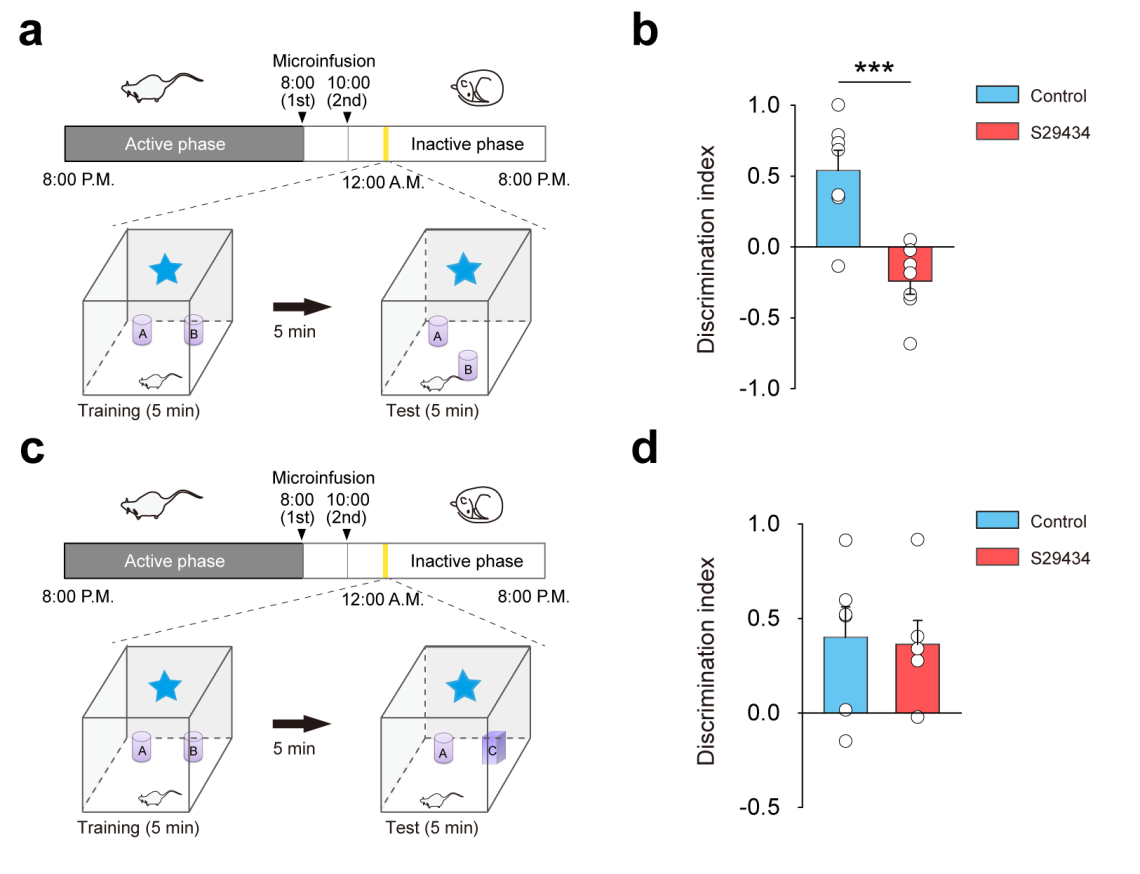


**Figure S12.** Blockade of MT_3_Rs during sleep selectively impairs the post-sleep spatial memory acquisition. a) Paradigm of behavior training and intervention of MT_3_Rs for exploring the role of MT_3_Rs in spatial memory acquisition. b) Blockade of MT_3_Rs during sleep in the MEC decreased the discrimination index in the spatial memory acquisition task (mean ± SEM). Student’s t-test, ****P* < 0.001, n = 7 rats. c) Paradigm of behavior training and intervention of MT_3_Rs for exploring the role of MT_3_Rs in object memory acquisition. d) Blockade of MT_3_Rs during sleep in the MEC did not affect the discrimination index in the object memory acquisition task (mean ± SEM). Student’s t-test, *P* = 0.861, n = 6 rats.


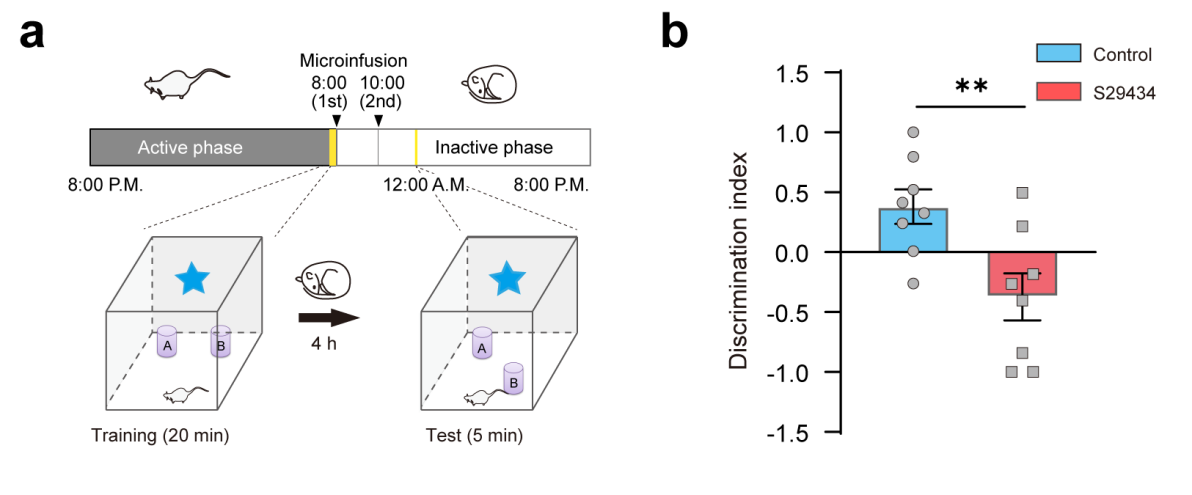


**Figure S13.** MT_3_R activation during sleep is indispensable for spatial memory consolidation. a) Paradigms of behavior training and intervention of MT_3_Rs for exploring the role of MT_3_Rs in spatial memory consolidation. b) Blockade of MT_3_Rs during sleep in the MEC impaired spatial memory consolidation (mean ± SEM). Student’s t-test, ***P* < 0.01, n = 8 rats


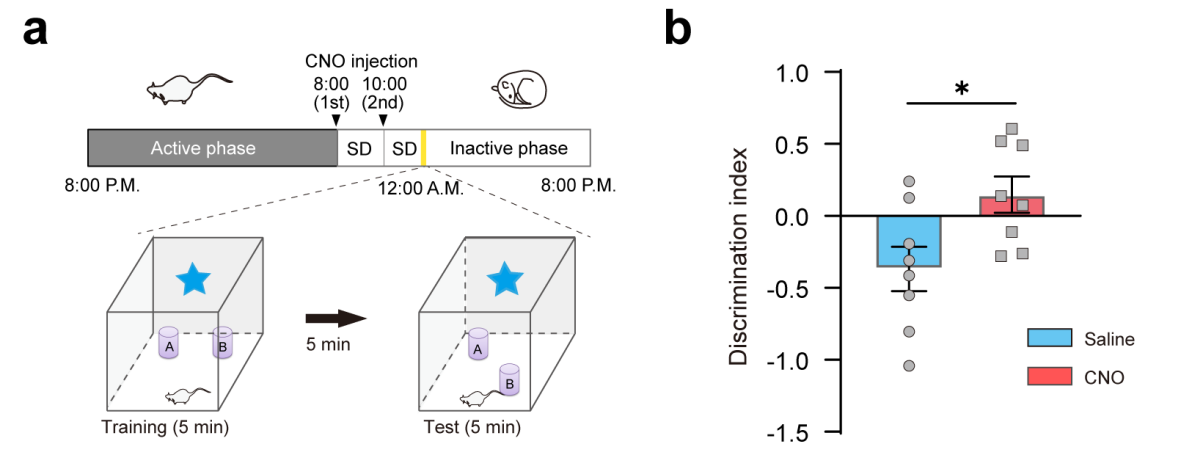


**Figure S14.** Inhibition of stellate neurons rescues sleep deprivation-induced spatial memory impairment. a) Paradigms of behavior training and chemogenetic inhibition of stellate neurons by injection of CNO during sleep deprivation. b) Chemogenetic inhibition of stellate neurons increased the discrimination index (mean ± SEM). Student’s t-test, **P* < 0.05, n = 8 rats.
